# Rational design of ASCT2 inhibitors using an integrated experimental-computational approach

**DOI:** 10.1073/pnas.2104093118

**Published:** 2021-09-10

**Authors:** Rachel-Ann A. Garibsingh, Elias Ndaru, Alisa A. Garaeva, Yueyue Shi, Laura Zielewicz, Paul Zakrepine, Massimiliano Bonomi, Dirk J. Slotboom, Cristina Paulino, Christof Grewer, Avner Schlessinger

**Affiliations:** ^a^Department of Pharmacological Sciences, Icahn School of Medicine at Mount Sinai, New York, NY 10029;; ^b^Department of Chemistry, Binghamton University, Binghamton, NY 13902;; ^c^Membrane Enzymology, Groningen Biomolecular Sciences and Biotechnology Institute, University of Groningen, 9747AG Groningen, The Netherlands;; ^d^Structural Bioinformatics Unit, Department of Structural Biology and Chemistry, CNRS UMR 3528, C3BI, CNRS USR 3756, Institut Pasteur, 75015 Paris, France;; ^e^Zernike Institute for Advanced Materials, University of Groningen, 9747AG Groningen, The Netherlands;; ^f^Structural Biology, Groningen Biomolecular Sciences and Biotechnology Institute, University of Groningen, 9747AG Groningen, The Netherlands

**Keywords:** solute carrier transporter, homology modeling, cryo-EM, membrane protein, MD simulations

## Abstract

ASCT2 (SLC1A5) is a sodium-dependent neutral amino acid transporter that controls amino acid homeostasis in peripheral tissues. In cancer, ASCT2 is up-regulated where it modulates intracellular glutamine levels, fueling cell proliferation. Nutrient deprivation via ASCT2 inhibition provides a potential strategy for cancer therapy. Here, we rationally designed stereospecific inhibitors exploiting specific subpockets in the substrate binding site using computational modeling and cryo-electron microscopy (cryo-EM). The final structures combined with molecular dynamics simulations reveal multiple pharmacologically relevant conformations in the ASCT2 binding site as well as a previously unknown mechanism of stereospecific inhibition. Furthermore, this integrated analysis guided the design of a series of unique ASCT2 inhibitors. Our results provide a framework for future development of cancer therapeutics targeting nutrient transport via ASCT2, as well as demonstrate the utility of combining computational modeling and cryo-EM for solute carrier ligand discovery.

## Significance

The glutamine transporter ASCT2 is an emerging therapeutic target for various cancer types. Here, we use an integrated computational and experimental approach to develop unique ASCT2 inhibitors targeting a conformational state useful for rational drug design. We apply computational chemistry tools such as molecular docking and molecular dynamics simulations, in combination with structure determination with cryo-electron microscopy and synthetic chemistry, to design multiple ASCT2 inhibitors. Our results reveal a unique mechanism of stereospecific inhibition of ASCT2 and highlight the utility of combining state-of-the-art computational and experimental approaches in characterizing challenging human membrane protein targets.

The Alanine-Serine-Cysteine Transporter 2 (SLC1A5, ASCT2) is a sodium-dependent transporter of neutral amino acids that belongs to solute carrier 1 (SLC1) family, which in humans includes excitatory amino acid transporters 1-5 (EAAT1-5) that transport glutamate, and neutral amino acid transporters ASCT1-2. Under physiological conditions, ASCT2 is expressed at low levels in various tissues, including the intestines, kidneys, liver, heart, placenta, and brain (1, 2). ASCT2 is emerging as an important therapeutic target for cancer and immunological disorders. In particular, ASCT2 is highly up-regulated by MYC in several cancer types such as triple negative breast cancer, prostate cancer, and melanoma (reviewed in ref.^3^), where it imports glutamine into cells that is utilized to build biomass and enhance proliferation via mTORC1 (4). Notably, inhibition of ASCT2 has been shown to reduce intracellular glutamine levels and, subsequently, tumor size in vivo (5). Recently, the role of ASCT2 in T cell differentiation and activation has been determined, expanding the relevance of ASCT2 to other T cell–based immune conditions (6).

Currently, there are no clinically relevant inhibitors of ASCT2, because ASCT2 pharmacology, like for many other SLC transporters, is poorly understood. A prerequisite for developing clinically relevant inhibitors for ASCT2 is the structural characterization of ASCT2’s distinct conformational states as well as its mode of interaction with inhibitors. Multiple structures of human SLC1 members and their prokaryotic homologs, combined with biophysical data, have demonstrated that these transporters operate using an elevator transport mechanism (7–12). In this mechanism, the transport domain with bound substrate moves perpendicularly to the membrane, while the static scaffold domain remains in place and constitutes the trimeric subunit interface. The conservation of the structure and mechanism across the SLC1 family has enabled the generation of high-quality models for human SLC1 members, capturing different snapshots of the transport cycle and aiding the development of novel inhibitors (13–17).

**Fig. 1.**
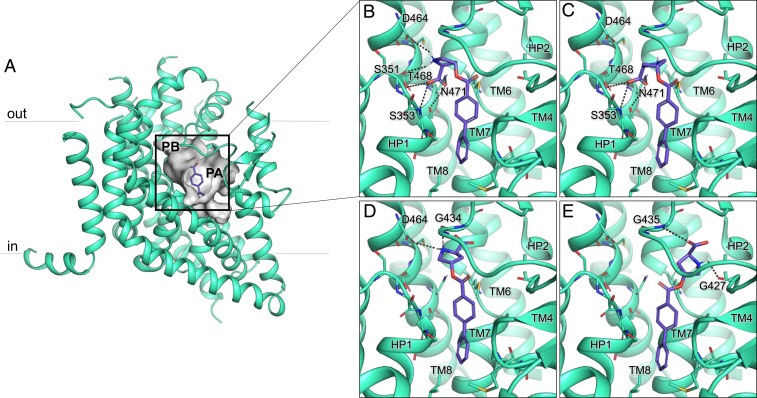
Predicted binding mode of ASCT2 inhibitors. (*A*) The outward-open homology model of ASCT2 based on the EAAT1 structure (PDB identification number: 5MJU) is shown as a green cartoon. The surface of the binding site is shown in gray, and subpockets A and B (PA and PB, respectively) are labeled. (*B*–*E*) The four ASCT2 inhibitors (purple) are cis and trans isomers of L- and D-proline derivatives (*Lc*-BPE, *Dc*-BPE, *Dt*-BPE, and *Lt*-BPE, respectively) that are predicted to bind a conformation-specific pocket accessible in the outward-open conformation, with HP2 in an open position. Key amino acids in the binding site are shown as sticks, where oxygen, nitrogen, and sulfur atoms are shown in red, blue, and yellow, respectively; hydrogen bonds are represented with gray dashes.

Recently determined cryo-electron microscopy (cryo-EM) structures of human ASCT2 (hASCT2) in various conformations have visualized distinct steps of the hASCT2 transport cycle for the first time (18–20). Specifically, the inward-occluded structure of hASCT2 with bound glutamine was solved first (19), confirming the hypotheses proposed based on homolog structures, particularly in terms of fold and transport mechanism. Subsequently, the inward-open structure of hASCT2 indicates that the transporter works using a single gate mechanism, in which hairpin loop 2 (HP2) controls access to the substrate binding site on both the intracellular and extracellular sides of the membrane. The structure also demonstrated that compounds can bind in the previously predicted subpocket region in the binding site, called pocket A (PA), that is revealed when the transporter is in an open conformation with HP2 hinged outward (Fig. 1) (18). Both the inward-open and outward-occluded structures of hASCT2 showed a putative cholesterol binding site at the interface between mobile transport and the static scaffold domains (18, 20).

Despite the progress of our understanding in the structure/function of hASCT2, a comprehensive description of hASCT2–ligand interactions on the molecular level, which is critical for drug development, is still lacking. Over the past few years, multiple small molecule competitive inhibitors have been developed for this protein, using both structure-based rational design and ligand-based approaches (reviewed in ref. 21) (*SI Appendix*, Fig. S1). For example, guided by homology modeling, we discovered and optimized a series of proline-based derivatives, as well as other chemical scaffolds, that inhibit ASCT2 with low micromolar (µM) potencies (14, 22, 23). Notably, these amino acid analog inhibitors, which have large hydrophobic groups in the sidechain, provided evidence that nonpolar interactions within the substrate binding site may be exploited to further increase potency. However, the low potency and selectivity of these inhibitors, combined with the lack of high-resolution visualization of their binding mode via experimentally solved structures, have hindered optimization efforts to develop clinically relevant ASCT2 compounds. Additionally, other human SLC1 members, hEAAT1 (SLC1A3) (24) and hEAAT2 (SLC1A2) (25), have been targeted by noncompetitive allosteric modulators, suggesting that similar strategies could also be applied to hASCT2. A potential limitation of such compounds is their increased lipophilicity and low aqueous solubility, hindering their utility in potential clinical applications.

Here, we rationally design unique inhibitors for hASCT2 by taking an integrated approach using computer-aided ligand design, organic synthesis, functional testing, molecular dynamics (MD) simulations, and structure determination with cryo-EM. We first refined the binding site of an outward-facing homology model of hASCT2 (26) based on an outward-open crystal structure of human EAAT1 (24) and used molecular docking, electrophysiology, and transport assays in proteoliposomes to develop and characterize potent hASCT2 inhibitors targeting a druggable subpocket in the substrate binding site. Next, we determined the cryo-EM structure of hASCT2 in complex with a potent inhibitor: *L*-*cis* hydroxyproline biphenyl ester (*Lc*-BPE) to reveal a mechanism of stereospecific inhibition. We then used MD simulations using the cryo-EM map as a restraint to identify conformations of hASCT2 and *Lc*-BPE that bind small molecules and are useful for drug design. These pharmacologically relevant states guided the design of multiple potent hASCT2 inhibitors, two of them with promising selectivity over EAATs. Finally, we discuss the pharmacological relevance of our results as well as the utility of our approach in guiding structure determination of challenging SLC targets.

## Results

### Rational Design of hASCT2 Inhibitors.

We have previously developed a series of hASCT2 inhibitors based on proline sulfonamide and sulfonic acid esters predicted to target a subpocket of the hASCT2 substrate binding site (pocket A, Fig. 1 and *SI Appendix*, Fig. S1), guided by homology models in the outward-open conformation (22). We found that molecules with a linker allowed compounds to dock into pocket A, and the inhibitor’s sidechain lipophilicity correlated with experimental inhibition constant (K_i_) values. Furthermore, the models correctly predicted the importance of a hydrophobic biphenyl group in the side chain for efficient inhibitor binding (22). Based on analysis of recent hASCT2 structures in the inward-open and the outward-occluded conformations (18–20), combined with docking of known ligands, we remodeled the hASCT2 binding site focusing on key side chains (i.e., S354, D464, and C467) (Fig. 1 and *Methods*). The resultant model performed better than any previously described cryo-EM structures or models of hASCT2 at enriching for known ligands as compared to a database of ligands and decoys. For example, the model obtained an area under the curve (AUC) of 94.3 as compared to AUC of 75 for the inward-open structure, suggesting the model represents an alternative ligand binding mode and ASCT2 conformation (22).

Additionally, our previously published proline-based ASCT2 inhibitors were based on the trans configuration of the stereocenter in position 4 of the proline ring (22, 23). However, we hypothesized that cis isomers would force the linker moiety of the proline-like scaffold to point toward the base of pocket A while maintaining critical polar interactions with hairpin 1 (HP1) and TM8 (e.g., with S353 and N471), typical of ASCT2 substrates and inhibitors (19, 20). Furthermore, to improve the solubility of the inhibitors while maintaining activity on ASCT2, we substituted the sulfonamide and sulfonic acid ester linkages with an ester linker, and, therefore, we synthesized four ester derivatives based on the 4-hydroxyproline amino acid scaffold in a first round of synthesized compounds. These four proline biphenyl esters (BPEs) are diastereomers with the L- (*L*c-BPE and *Lt*-BPE) and D-configuration (*Dc-*BPE and *Dt*-BPE) at the α-amino acid carbon. We then used molecular docking and estimated the free energy of binding using molecular mechanics/generalized born surface area (MM-GBSA) to assess binding of the new ligand series (*SI Appendix, Supplementary Materials and Methods* and Table 1). Interestingly, both docking scores and MM-GBSA calculations of cis and trans isomers of the L and D compounds (Fig. 1 and Table 1) predict that cis compounds are more potent than their trans counterparts. We also observe that the L-amino acid scaffold is preferred to the D-amino acid, in agreement with a previous report (27).

**Table 1.** Initial tool compounds developed in this study (first series)

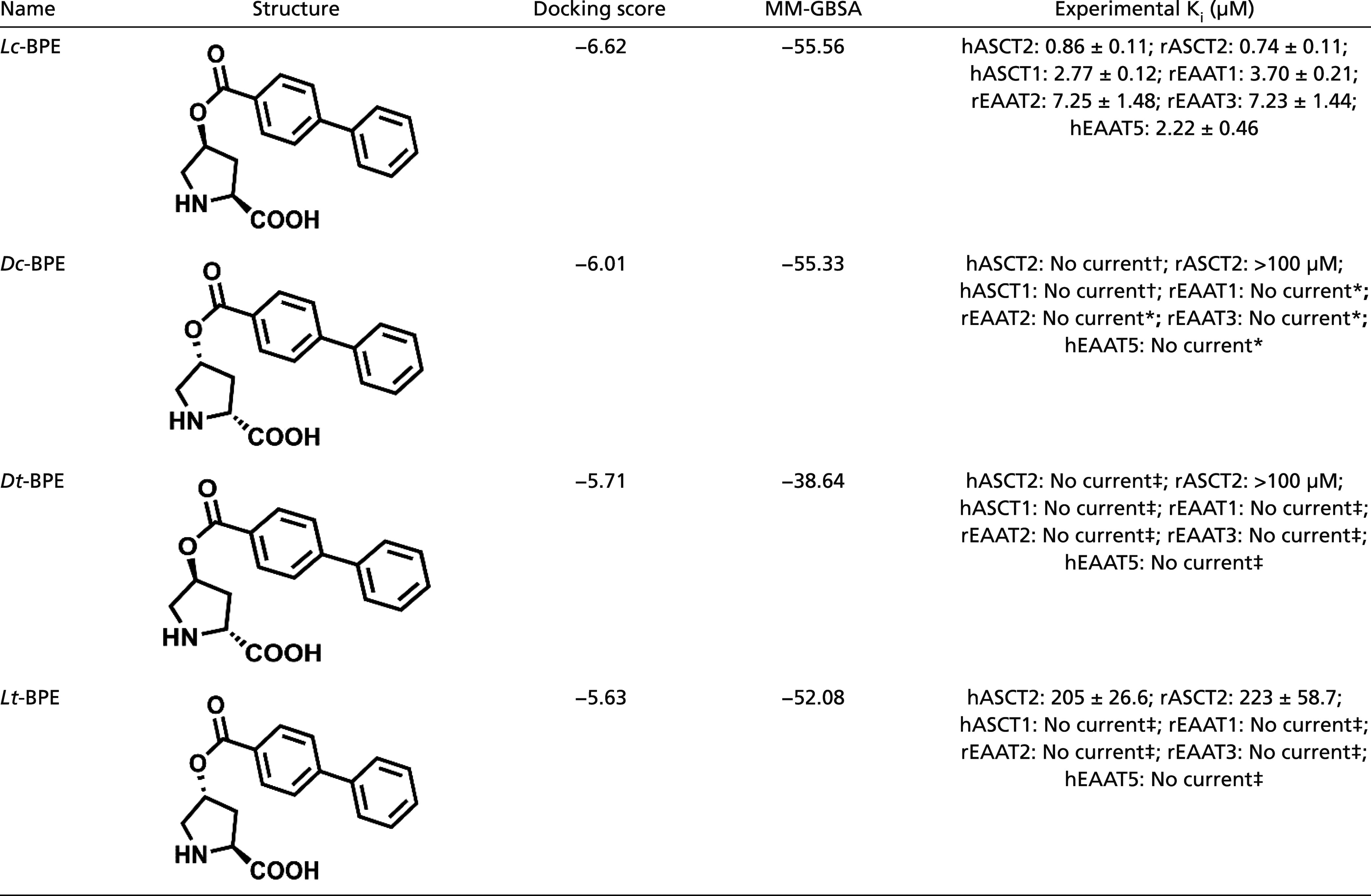

“Name” marks the compound name. Names include *Lc*-BPE, *Dc*-BPE, *Dt*-BPE, and *Lt*-BPE. “Structure” corresponds to the two-dimensional structure of the compound drawn by PerkinElmer ChemDraw. “Docking score” marks the docking score of the top scoring pose using Glide. “Experimental K_i_” marks the experimental K_i_ values (obtained from electrophysiological experiments). “MM-GBSA” corresponds to the predicted free energy of binding. “No current” indicates that no outward current (i.e., inhibition of anion leak current) was observed in these experiments. This means that the compound either does not bind or binds but is unable to block the leak anion current.

* The highest inhibitor concentration tested in µM, where 20 ≤ * ≤ 50.

^†^ The highest inhibitor concentration tested in µM, where 100 ≤ ^†^ ≤ 400.

^‡^ The highest inhibitor concentration tested in µM, where 500 ≤ ^‡^ ≤ 1,000.

### *Lc*-BPE Inhibitory Activity with Submicromolar Potency.

**Fig. 2.**
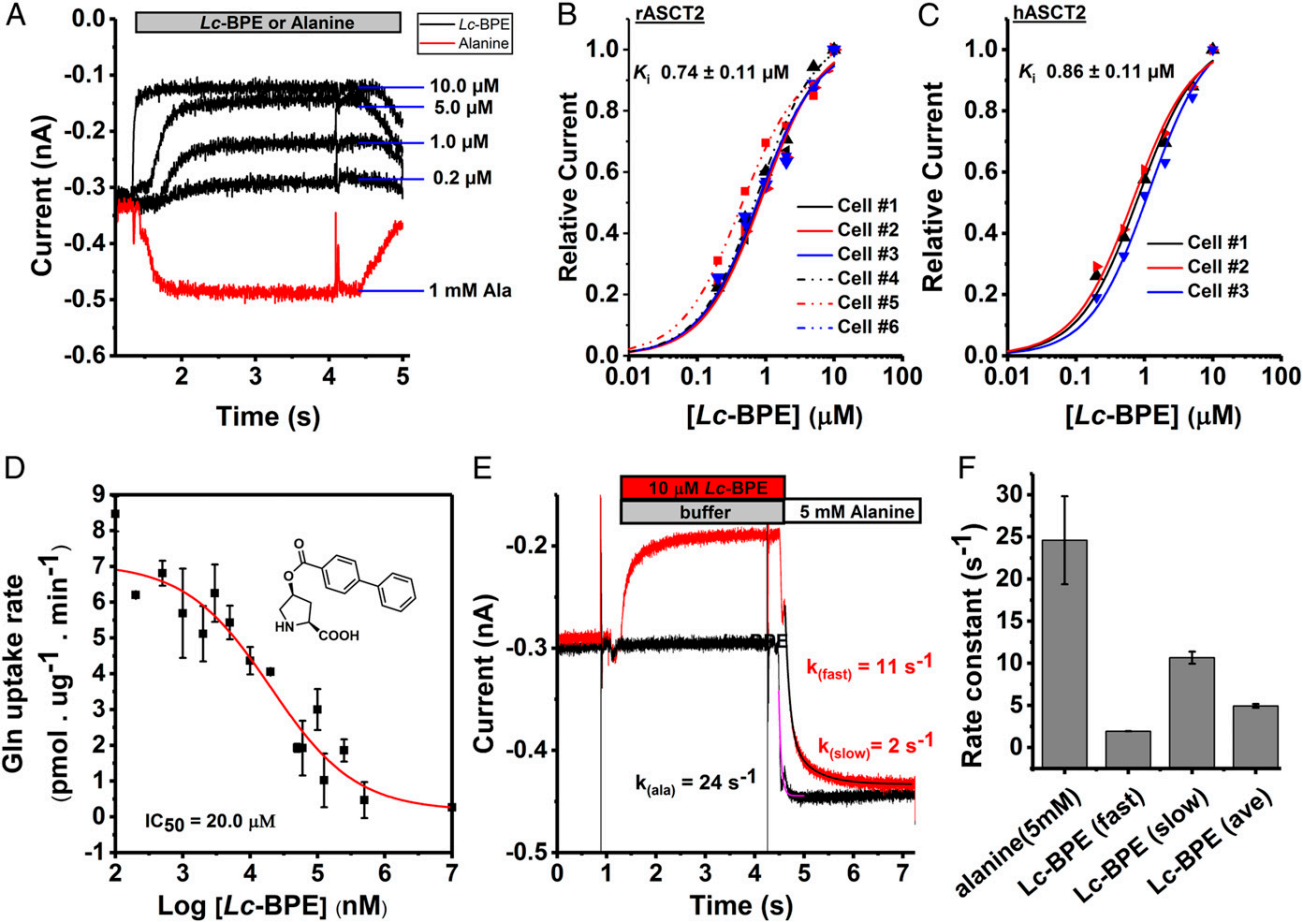
*Lc*-BPE is a potent inhibitor of ASCT2. (*A*) Electrophysiological recordings obtained after application of alanine (red trace) and increasing concentrations of *Lc*-BPE (black traces). The times of application of alanine or *Lc*-BPE are shown by a gray bar (top). (*B* and *C*) *Lc*-BPE inhibitor dose–response relationships for rASCT2 and hASCT2, respectively. The solid and dashed lines represent the best fits to a Michaelis–Menten-like equation with an average apparent *K*_i_ of 0.74 ± 0.11 µM (rASCT2) and 0.86 ± 0.11 µM (hASCT2). Currents were normalized to the current recorded after application of 10 µM inhibitor concentration. (*D*) Inhibition of glutamine uptake (5 µM external concentration) in the presence of varying concentration of *Lc-*BPE and 5 µM glutamine measured in proteoliposomes reconstituted with purified hASCT2. A half-maximum inhibitory concentration (IC_50_) of 20.0 µM (black line) is calculated from five biologically independent measurements. Errors bars represent SEM from at least two biologically independent measurements. (*E*) Current recorded after rapid solution exchange from extracellular buffer (control, gray bar, black trace) to a solution containing buffer + 5 mM alanine (white bar). For the red trace, the cell was preincubated with 10 µM *Lc*-BPE (red bar, red trace) followed by rapid application of 5 mM alanine. All current recordings were performed at 0 mV in the presence of 130 mM NaSCN internal and 140 mM NaCl external (homoexchange) solutions. (*F*) Average rate constants, k_obs_, for data shown in *E*. Rate constant of the current rise when 5 mM alanine is rapidly applied is shown in pink (24.6 ± 5.2 s^−1^, control). The rate constants in the presence of preapplied *Lc*-BPE followed by 5 mM alanine application are 1.9 ± 0.1 s^−1^ (fast) and 10.7 ± 0.7 s^−1^ (slow) with a mean of 4.9 ± 0.2 s^−1^ (single-exponential fit). All current recordings were performed at 0 mV in the presence of 130 mM NaSCN internal and 140 mM NaCl external (homoexchange) solutions.

The four initially synthesized compounds were tested for inhibitory activity in rat ASCT2 (rASCT2) with electrophysiology. rASCT2 was used initially because it exhibits similar substrate and inhibitor specificity to that of the human ASCT2 while having better expression in HEK293 cells than that of hASCT2 (13, 21, 22, 28). Notably, it is known that competitive inhibitors block a tonic ASCT2 leak anion conductance (28), which results in the inhibition of inward current in the presence of intracellular permeable thiocyanate (SCN^–^), see apparent outward current in Fig. 2*A* and *SI Appendix*, Fig. S2). In contrast, transported substrates activate the anion conductance (29), resulting in an inward current caused by SCN^–^ outflow (Fig. 2*A* and *SI Appendix*, Fig. S2). The leak anion conductance was used as an electrophysiological measure of ASCT2 activity because ASCT2 steady state amino acid exchange is electroneutral and thus does not catalyze transport current. It was previously shown that the anion conductance correlates with transport activity for ASCT2 (28, 30) and other SLC1 members (31). The inhibition of the anion leak current was dose dependent and could be fitted to a Michaelis–Menten-type equation to yield an apparent K_i_ (Fig. 2*B* and *SI Appendix*, Fig. S3*B*). In contrast to the L-based diastereomers (K_i_ of 0.74 ± 0.11 µM for *Lc*-BPE and 232 ± 44 µM for *Lt*-BPE in rASCT2), the esters based on the D-amino acid scaffold did not show significant inhibition of the leak anion current, indicating that they are not inhibitors of rASCT2 within the concentration range tested (up to 200 µM, Table 1). Further, the two L-isomers inhibited leak anion current, with *Lc*-BPE showing the strongest apparent outward current (Table 1 and *SI Appendix*, Fig. S2 *A*–*C*). Similar experiments were performed using hASCT2 yielding a K_i_ of 0.86 ± 0.11 µM for *Lc*-BPE (Fig. 2*B*) and 205 ± 27 µM for *Lt*-BPE. Notably, the experimental K_i_s of these newly synthesized compounds agreed with the computational predictions (Table 1). For example, *Lc*-BPE had the best docking score and estimated binding affinity, and its docking pose placed the ligand in pocket A while maintaining the critical interactions with HP1 and TM8, as expected from previously published structures of the human ASCT2 (18–20). Taken together, these results support the hypothesis that stereochemistry is important for stronger binding with proline scaffold inhibitors with “long,” bulky substituents.

We also tested the specificity of *Lc*-BPE for ASCT2 over other SLC1 family members (ASCT1, EAAT1-3, and EAAT5). *Lc*-BPE showed inhibitory activity in all SLC1 family members tested, including EAATs, as predicted from the high conservation of pocket A among SLC1 members (21, 22, 32). However, the apparent affinity of human ASCT1 (hASCT1) for *Lc*-BPE was ∼3.8-fold lower than that of rASCT2 (*SI Appendix*, Fig. S3 *B* and *C*), showing slight preference for ASCT2. For the EAATs, the highest affinity interaction of *Lc*-BPE was with hEAAT5, K_i_ of 2.22 ± 0.46 µM, which was ∼3-fold higher K_i_ than rASCT2, while interaction with EAAT1, EAAT2, and EAAT3 was weaker (*SI Appendix*, Fig. S3). This result was not unexpected, because prototypical EAAT inhibitors such as TBOA (33), which also have hydrophobic groups in the side chain that interact with pocket A, are also not entirely selective.

### *Lc*-BPE Is a Competitive Inhibitor.

Our computational models predicted *Lc*-BPE as a competitive inhibitor targeting the substrate binding site. To test this prediction, we performed electrophysiological competition experiments in the presence of varying concentrations of the substrate, L-alanine. The results were consistent with a competitive inhibition mechanism (*SI Appendix*, Fig. S4). At low inhibitor concentrations, the alanine-induced currents were inward, as expected from transported substrates, indicative of the inability of *Lc*-BPE to displace alanine from the binding site. However, as inhibitor concentration increased, the currents became outwardly directed in a dose-dependent manner, which marked the displacement of alanine by the inhibitor (*SI Appendix*, Fig. S5). The apparent K_i_ increased as a function of the alanine concentration, as anticipated for a competitive mechanism (*SI Appendix*, Figs. S5 and S6). In addition to currents generated by L-alanine as a substrate, *Lc*-BPE also inhibited L-glutamine–induced currents in a similar manner (*SI Appendix*, Fig. S7), corroborating these observations. We further tested the inhibitory activity of *Lc*-BPE in a cell-free transport assay in proteoliposomes, where purified and reconstituted hASCT2 catalyzes the exchange of intracellular nonlabeled L-glutamine with extracellular radioactive-labeled L-glutamine. We observed a dose-dependent inhibition of glutamine transport when *Lc*-BPE was added externally, which confirms that this compound is an hASCT2 inhibitor with a half-maximum inhibitory concentration (IC_50_) value of 20 µM in the presence of 5 µM glutamine (Fig. 2*D*).

### Inhibitor Dissociation Kinetics.

Due to the potency of *Lc*-BPE, we hypothesized that the inhibitor dissociates slowly from the ASCT2 binding site. To test this, we measured the dissociation kinetics of *Lc*-BPE using a ligand displacement approach. First, rASCT2-expressing cells were preincubated with a saturating solution of *Lc*-BPE (10 µM), and subsequently the inhibitor was displaced using a supersaturating concentration of the substrate L-alanine (5 mM) (Fig. 2 *E* and *F*). It is expected that the inhibitor would have to dissociate before alanine could bind and activate the inward anion current. Consistent with this expectation, activation of the anion current by alanine was slowed significantly after *Lc*-BPE preincubation by more than sixfold. The rate constant for *Lc-*BPE dissociation was estimated as 2.16 s^−1^, indicative of a long residence time of the compound in the rASCT2 binding site, in the range of 500 ms.

### Cryo-EM Structure of hASCT2-*Lc*-BPE.

**Fig. 3.**
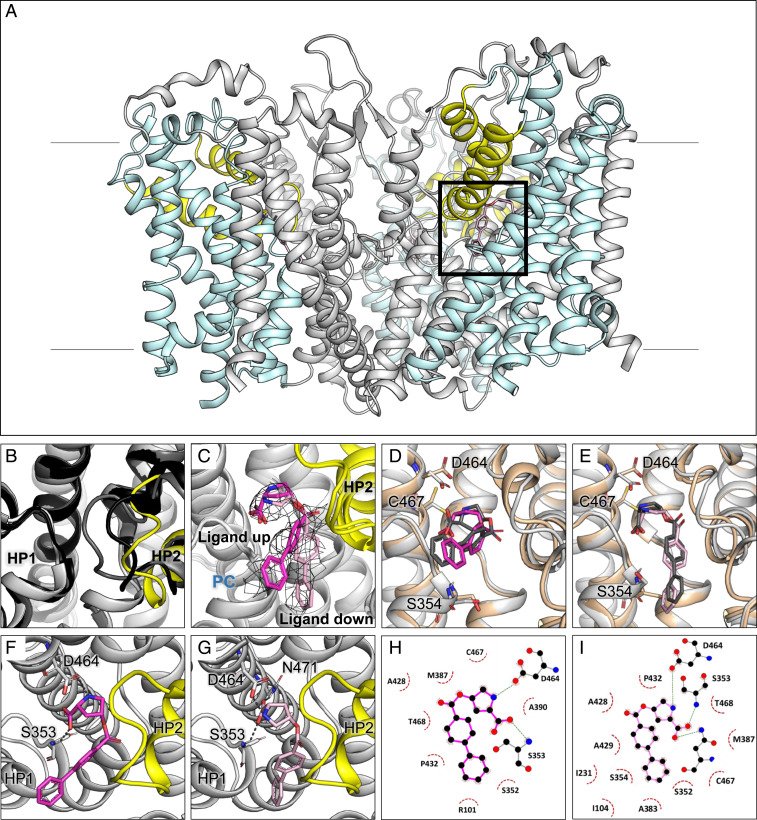
Structural basis for ASCT2 inhibition by *Lc*-BPE. (*A*) Cryo-EM structure of the outward-open ASCT2 trimer in complex with *Lc*-BPE. The scaffold and transport domains are depicted in gray and blue ribbons, respectively, with HP2 shown in yellow. Lines show the approximate location of the membrane boundaries. The binding site is roughly outlined with a box. (*B*) Superposition of the glutamine-bound outward-occluded (6MPB; gray), apo outward-open (6MP6; black), and outward-open structure in complex with *Lc*-BPE (yellow) of ASCT2 with a tentative model of HP2, highlighting the HP2 position for the different conformations. (*C*) Zoomed-in view of the substrate binding site of ASCT2 showing two possible modes of *Lc*-BPE binding. Mesh represents density in the cryo-EM map. Dark pink and light pink ligands represent the ligand-up and ligand-down conformations, respectively. PC is short for pocket C. (*D* and *E*) Progressive model building and refinement of the binding site. Superposition of initial (tan) and refined (light gray) structures with remodeled residues. (*D*) *Lc*-BPE in the refined ligand-up structure is dark pink, and (*E*) *Lc-*BPE in the refined ligand-down structure is light pink. (*F* and *G*) Potential hydrogen bonds between inhibitor and protein for each ligand conformation shown as dashed lines. In the ligand-up conformation (*F*), the distal phenyl ring of the ligand interacts with previously unknown subpocket C. (*H* and *I*) Two-dimensional ligand interaction plot visualized with LigPlot+ (46) of (*H*) ligand-up and (*I*) ligand-down conformations. Hydrogen bonds are represented as green dashes, and residues making hydrophobic interactions are marked with red dashes and labeled.

While ASCT2 homology models were useful to identify initial compounds, an experimentally determined structure of ASCT2 in complex with the ligand is needed to better describe the molecular interactions between ASCT2 and *Lc*-BPE as well as to aid further inhibitor optimization of our initial ASCT2 inhibitor. We determined the structure of hASCT2, reconstituted into lipid nanodiscs and in the presence of the inhibitor *Lc*-BPE, at 3.4 Å resolution using single-particle cryo-EM (Fig. 3, *SI Appendix*, Fig. S8, and Table 2). The protein adopts a single symmetrical state with all three protomers in the same outward-facing conformation, in which *Lc*-BPE precludes HP2 from closing. The structure confirmed our computational and experimental findings that *Lc*-BPE is a competitive inhibitor of the substrate binding site. Interestingly, the inhibitor-bound structure captures a pharmacologically relevant outward-open conformation, indicating that the inhibitor could bind to ASCT2 from the extracellular side, thereby not requiring a mechanism to cross the cell membrane.

**Table 2.** Cryo-EM data collection, refinement, and validation statistics

	*Lc*-BPE-bound ASCT2, position up (EMD-12142, PDB 7BCQ)	*Lc*-BPE-bound ASCT2, position down (EMD-12142, PDB 7BCS)
Data collection and processing		
Magnification	49,407	
Voltage (kV)	200	
Electron exposure (e–/Å^2^)	53	
Defocus range (μm)	−0.9 to −1.9	
Pixel size (Å)	1.012	
Symmetry imposed	C3	
Initial particle images (No.)	1,801,520	
Final particle images (No.)	300,899	
Map resolution (Å)	3.43	
FSC threshold	0.143	
Map resolution range (Å)	3.3 to 4.4	
Refinement		
Initial model used	PDB 6MPB	
Model resolution (Å) (0.5 FSC threshold)	3.4	
Model resolution range (Å)	15 to 3.4	
Map sharpening *B* factor (Å^2^)	−189	
Model composition		
Nonhydrogen atoms	9,861	9,861
Protein residues	1,326	1,326
Ligands	3	3
*B* factors (Å^2^)		
Protein	40.14	37.78
Ligand	36.79	30.07
R.m.s. deviations		
Bond lengths (Å)	0.010	0.010
Bond angles (°)	1.062	1.074
Validation		
MolProbity score	1.49	1.54
Clashscore	3.22	3.97
Poor rotamers (%)	0.00	0.00
Ramachandran plot		
Favored (%)	94.55	94.77
Allowed (%)	5.45	5.23
Disallowed (%)	0.00	0.00

On closer inspection, L*c*-BPE overlaps with the region that HP2 occupies in the substrate-bound occluded and substrate-free states. Despite being poorly resolved, we tentatively modeled the HP2 loop, which is likely further hinged away than seen in the other hASCT2 outward-open conformation (Fig. 3*B* and *SI Appendix*, Fig. S8*H*) (20). The displacement of HP2 appears to be a consequence of inhibitor binding, suggesting that the HP2 gate is flexible and can potentially move even further away to accommodate bulkier inhibitors. In further support of this hypothesis, the HP2 in this structure (*SI Appendix*, Fig. S8*H*), as well as in other hASCT2 structures, is less resolved, indicative of structural flexibility (18–20). This observation offers a strategy to improve inhibitor potency by designing larger inhibitors to specifically block the gating mechanism of the transporter. In addition, we observed a strong unassigned density in the binding site, which overlaps with the density of glutamine in the previous substrate-bound hASCT2 structures (19, 20). This density further protruded outside the region occupied by glutamine in the previous structures in two directions, indicative of multiple ligand binding modes. Building *Lc*-BPE along both of these cryo-EM densities resulted in two binding modes, with the ligand occupying either an “up” or a “down” orientation (Fig. 3*C*). While the “ligand-down” orientation resembled the one predicted from docking studies, the “ligand-up” orientation represents an empirically determined binding mode. Here, *Lc*-BPE is located in a space between HP1 and TM2, thereby occupying an uncharacterized subpocket in the substrate binding site, which we refer to as pocket C. Additional rounds of cryo-EM model refinement were guided by a closer inspection of the cryo-EM density as well as by a detailed comparison of the initial fitted cryo-EM model with the ligand docked homology model and other available SLC1 structures (*SI Appendix*, Fig. S9 *A*–*C*). This led to two distinct rearrangements of S354, C467, and D464 in the binding site, required to accommodate a ligand-up or a ligand-down binding mode (Fig. 3 *D*–*G*). Indeed, the refined cryo-EM structure for the ligand-down ligand binding conformation obtained an improved enrichment score (AUC of 84.6 and logAUC 44.9 compared to AUC 69.5 and logAUC 32.4), supporting the remodeling (*SI Appendix*, Fig. S9*D*). Overall, these results suggest the existence of two inhibitor binding modes, ligand up and ligand down.

Concurrent with previous structures of hASCT2 and other homolog structures with bound ligand (8, 24, 34), we observe conserved critical polar contacts with the backbone of S353 in HP1 in both ligand-up and ligand-down conformations of the refined structures (Fig. 3 *F*–*I*). In general, ligand-up *Lc*-BPE is coordinated by binding site residues S353 and D464, while ligand-down *Lc*-BPE is coordinated by S353, D464, and N471 (Fig. 3 *F*–*I*). Further analysis of the two potential binding modes showed that the ligand-down state has more favorable hydrophobic interaction at the base of pocket A compared to the ligand-up state (Fig. 3 *H* and *I*).

### Characterization of Multiple Binding Modes with MD Simulations.

Areas of lower resolution in cryo-EM maps can be the result of averaging multiple different conformations during image processing. To investigate the heterogeneity in the hASCT2 and *Lc*-BPE conformations and explore the possibility of multiple ligand binding modes, we used meta-inference MD simulations (35) to model an ensemble of conformations consistent with the cryo-EM data (36). In meta-inference, the molecular mechanics force field used in standard MD is augmented by spatial restraints that enforce the agreement of an ensemble of replicas of the system with the cryo-EM density. Using a Bayesian inference framework, this approach accounts for the simultaneous presence of structural heterogeneity, data ensemble averaging, and variable level of noise in different areas of the experimental map. Meta-inference has recently been successfully used to characterize the structural heterogeneity of the N-terminal gating region of the ClpP protease (37) and the effect of acetylation on the dynamics of the K40 loop in α-tubulin (38).

**Fig. 4.**
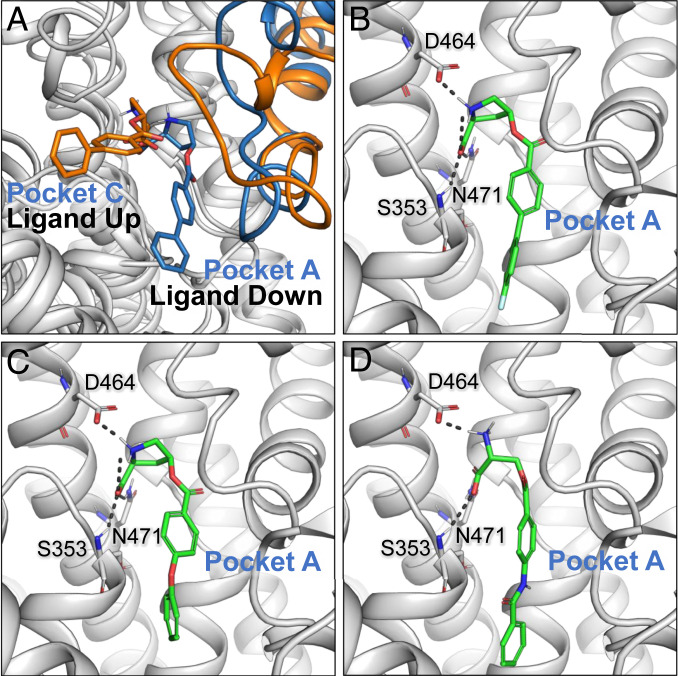
Pharmacological relevance of multiple ligand binding modes. (*A*) Clusters of ligand conformations obtained with meta-inference MD simulations reveal multiple ligand binding modes. Orange and blue, respectively, represent the ligand-up and ligand-down conformations of *Lc*-BPE and HP2. (*B–D*) Molecular docking of second-generation inhibitors to hASCT2 structures with the inhibitors colored in green. *B*–*D* are ERA-4, ERA-16, and ERA-21, respectively.

The ensemble of models obtained with meta-inference was classified based on the positions of the ligand atoms and those of the surrounding protein residues (*SI Appendix*). This analysis indeed suggests that the observed cryo-EM map is the result of an equilibrium of multiple conformations in which the ligand occupies distinct poses and flexible HP2 loop conformations, as anticipated. We observed six clusters in total, which we further grouped into three distinct groups based on visual analysis (*SI Appendix*, Fig. S10). These groups support the presence of multiple pharmacologically relevant conformations. Interestingly, the two most populated groups are highly similar to the ligand-up and ligand-down structure and homology model, with the third group (5%) being unlikely to be replicated under physiological conditions (*SI Appendix*, Fig. S10 and Fig. 4*A*). This analysis also supports the idea that HP2 is highly flexible and that its position is dependent on the bound inhibitor. We quantified the agreement of the cryo-EM structure and the meta-inference ensemble with the observed cryo-EM density using the cross-correlation between the experimental and simulated maps. In the former case, the simulated map was calculated on the individual cryo-EM model; in the latter case, the simulated map was averaged over all members of the meta-inference ensemble. The cross-correlation was computed on the entire experimental grid (CC_box) and was equal to 0.403 and 0.684 in the case of the individual cryo-EM structure and of the meta-inference ensemble, respectively. Overall, the meta-inference ensemble provided a better fit of the density map compared to the cryo-EM structure prior to B-factor refinement, without compromising the stereochemical quality of the models (*SI Appendix*, Fig. S10*D*).

### Ligand Discovery of Conformation-Specific Inhibitors.

We tested the pharmacological relevance of the two different binding modes of *Lc*-BPE and the model for proposed stereospecificity by developing additional ASCT2 inhibitors based on both 4-hydroxyproline and serine scaffolds. We docked 14 analogs to the substrate binding sites of both ligand-up and ligand-down structures. The compounds generally scored better when docked in the ligand-down structure. Furthermore, any compound that did not dock in a ligand-up or ligand-down (“other”) pose showed poor or no activity, with the exception of ERA-11 (*SI Appendix*, Table S1). Remarkably, the predicted binding mode of the ligands agreed with their activity (*SI Appendix*, Table S1 and Fig. 4). Proline scaffold compounds that docked in a ligand-down pose generally showed inhibitory activity in the low µM range or better (*SI Appendix*, Table S1 and Fig. 4 *B* and *C*). In addition, by increasing the substituent length to access a larger fraction of pocket A (ligand down), we improved compound potency as shown with ERA-4 with the addition of fluorine at the para position of the biphenyl side chain (K_i_ = 0.74 ± 0.09 µM). There were no compounds that scored better in the ligand-up structure that occupied a ligand-up pose. Overall, while these data are consistent with the pharmacological relevance of both ligand-up and ligand-down conformations, it suggests that the ligand-down conformation may be more useful for the design of compounds with consistent structure activity relationship (SAR).

In addition, a few other observations are relevant for determining structure activity relationships of the side chain substituent: 1) increased hydrophobicity results in potent binding to rASCT2 but less so in hASCT2 (compounds ERA-4 and ERA-5); 2) connection of the biphenyl group in ortho position results in an inactive compound (ERA-3); 3) branching of the side chain next to the ester position led to a compound with no activity (ERA29); and 4) preference of cis versus trans stereochemistry was confirmed with the benzophenone substituent (ERA-8 and ERA-35).

To further this analysis, we explored three serine esters. As expected from previous observations (13, 28), the compound based on the serine scaffold did not reach submicromolar affinity (*SI Appendix*, Table S1 and Fig. 4*D*). The potency of serine was considerably reduced compared to a hydroxyproline ester with comparable sidechains (ERA-11 and ERA-21). The IC_50_ obtained from glutamine uptake inhibition in proteoliposomes performed on a selection of compounds, namely ERA-8, ERA-21, and ERA-35, reiterate a similar inhibitor efficacy (*SI Appendix*, Table S1).

We also tested specificity of the whole series of compounds with respect to ASCT1, ASCT2, EAAT1-3, and EAAT5. Most compounds displayed little preference for ASCTs over EAATs. This was somewhat expected based on the similarity of the side chains to known EAAT inhibitors as well as the conservation of pocket A among the SLC1 members (*SI Appendix*, Fig. S11). Two notable exceptions are ERA-9, which has a hydroxy group in the side chain, and ERA-11. ERA-9 showed moderate selectivity for hASCT2 over EAATs, with a sevenfold higher affinity for hASCT2 (K_i_ = 5.7 ± 1.3 µM) versus rEAAT1 (K_i_ = 39.5 ± 19.1 µM), while ERA-11 was threefold selective for hASCT2 over rEAAT1 (*SI Appendix*, Table S1). No inhibitory activity was detected with ERA-9 for the other EAATs. In addition, affinity for hASCT1 was also low, with a K_i_ value of 43.0 ± 13.4 µM. These results suggest that development of selective ASCT2 inhibitors will be a challenge but that potential hydrophilic interaction in the side chain may provide an avenue toward more selective compounds.

## Discussion

In this study, we combined computational modeling and experimental testing to describe the molecular basis for ASCT2 inhibition. Three key findings emerge from our work. First, we developed multiple ASCT2 inhibitors that provide unique chemical tools for further studying the role of ASCT2 in cancer and other diseases. For example, *Lc*-BPE is a competitive, stereoselective inhibitor with submicromolar potency. *Lc*-BPE was shown to bind the substrate binding site of ASCT2 (Fig. 2 and Table 1). Second, a cryo-EM complex structure of hASCT2 with *Lc*-BPE revealed a unique movement of HP2 in the binding site of the outward-open conformation. This result describes the mechanism by which ASCT2 inhibitors achieve their stereospecificity and provides a framework for developing future clinically active, ASCT2-selective compounds. Third, our computational analysis, including iterative homology modeling, model refinement, and MD simulations using the cryo-EM density as a restraint, revealed distinct conformations of the ligand and ASCT2 binding site (Figs. 3*C* and 4). This finding guided optimization of initial hits, suggesting that a similar approach could be applied to design and characterize inhibitors for other challenging human SLC targets in different conformations. We discuss each of these three findings in turn.

### Chemical Tools to Characterize ASCT2.

A chemical tool is a molecule capable of modulating the function of a protein, enabling researchers to ask mechanistic questions about its molecular target with various experimental systems, including biochemical, cellular, or in vivo approaches. Notably, a useful chemical probe needs to be validated experimentally using orthogonal assays (39). Although the SLC1 family has been established as an important family of druggable proteins, the SLC1 members, including ASCT2, currently suffer from a limited number of chemical probes to help decipher their role in disease. Here, we developed multiple ASCT2 inhibitors with unique chemical scaffolds that make previously unknown interactions with ASCT2. For example, the isomer with the highest apparent affinity from the first compound series, *Lc*-BPE, was validated using electrophysiological, cellular, biochemical, and structural approaches directly showing target engagement and confirming a competitive stereoselective inhibition mechanism. As demonstrated for *Lc*-BPE and the inhibitors designed based on the hASCT2-*Lc*-BPE structure, compounds make specific interactions with S353 and D464, which have been shown to be critical for ASCT2 substrate binding (18–20) and interact with a conserved pocket across all human SLC1 members (pocket A) (Fig. 3 *D*–*I* and *SI Appendix*, Fig. S11). It should be noted that compounds *Lc*-BPE (K_i_ of 0.86 ± 0.11 µM) and ERA-4 (K_i_ of 0.74 ± 0.09 µM) are inhibitors for ASCTs with a submicromolar affinity, providing a major step toward inhibitors with high affinity ASCT2 interaction.

Encouragingly, as expected from their predicted binding poses, most compounds developed in this study are not strongly selective for ASCT2; however, ERA-9 and ERA-11 exhibit promising selectivity (*SI Appendix*, Table S1) as compared to the EAATs, providing an excellent starting point for designing ASCT2-specific compounds that can potentially avoid neurotoxicity resulting from binding to EAATs. Expanding SAR on these two compounds to target unique residues in pocket B of this protein (e.g., C467; Fig. 1 and *SI Appendix*, Fig. S11) may lead to compounds with clinical relevance.

### Atomic Description of Stereoselective Inhibition of ASCT2.

Despite progress in methodologies for structure determination of membrane proteins, only a few human SLC transporter structures have been determined at high resolution (32). Furthermore, SLCs, including ASCT2, are highly dynamic, and visualization of different conformational states that are associated with transport or inhibition can be useful for the development of unique substrates and inhibitors. It was recently shown that transporters can adopt multiple “active” conformations that are associated with transport or inhibition, in which the transporter interacts uniquely with different substrates or inhibitors in the binding pocket (40). These different substates can have varying relevance for rational drug design.

Here, we report the cryo-EM structure of hASCT2 bound to a potent inhibitor, *Lc*-BPE, in a unique outward-facing state and demonstrate its usefulness for compound design as well as our understanding of the mechanism of the proline scaffold stereospecificity. The ester derivatives based on the 4-hydroxyproline amino acid moiety in *Lc*-BPE are constrained to adopt ligand-down conformation, different from its stereoisomers. The cis configuration of *Lc*-BPE allowed the compound to make key interactions with residues in HP1 (S353) and with residues in TM8 simultaneously, a key feature of ASCT2-ligand binding, while facilitating hydrophobic interactions in pocket A via the linker region. The structure also revealed that *Lc*-BPE binding displaces HP2 in hASCT2 (Fig. 3*B*). The flexibility of HP2 is further supported by the observation that HP2 is less resolved in this structure (*SI Appendix*, Fig. S8*H*), similar to other hASCT2 structures (18–20). While the structure guided the development of multiple potent inhibitors, it also suggests that HP2 can potentially move even further away to accommodate bulkier inhibitors, leaving room for further optimization of compounds.

### Integrated Approach Identifies Multiple Pharmacologically Relevant States.

While cryo-EM has become the most dominant technology for structure determination of human SLC transporters (41), hybrid strategies involving computational methods can efficiently sample and visualize SLC transporters in different conformations and expedite structure determination with cryo-EM (42, 43). Recently, the combination of different sampling and refinement approaches with ligand docking has aided the design of small molecule ligands, particularly, for challenging membrane protein targets such as G protein-coupled receptors (GPCRs) and transient receptor potential (TRP) channels (18, 32, 44, 45). In this work, the integration of multiple computational methods throughout the structure determination process aided the characterization of ASCT2–ligand interactions. First, the hASCT2 homology model based on the human EAAT1 structure 1) obtained high ASCT2 ligand enrichment (*SI Appendix*, Fig. S9*D*), 2) exhibited high structural similarity to the later experimentally solved cryo-EM structure (*SI Appendix*, Fig. S9 *A*–*C*), and 3) supported precise cryo-EM model refinement (*SI Appendix*, Fig. S9 *C* and *D*). In combination, two ligand binding modes, ligand-down and ligand-up, were obtained, revealing distinct orientations of S354, D464, and C467 that are critical for *Lc*-BPE binding and substrate specificity (18–20). This result highlights that homology modeling can be a useful complementary approach to empirical structure determination for the design of conformation-specific inhibitors.

MD simulations, with the cryo-EM density map as a restraint, revealed multiple potential hASCT2-*Lc*-BPE configurations that can be further grouped into three putative positions of the ligand (Figs. 3*C* and 4 and *SI Appendix*, Fig. S10). The vast majority of them resembled the empirically determined ligand-up and ligand-down conformations, increasing our confidence in the approach. This finding reveals heterogeneity in ligand recognition in the SLC1 family. Furthermore, the relevance of the different conformations for compound development is demonstrated by the design of second-generation compounds (*SI Appendix*, Table S1). We identified the ligand-down state as the key pharmacological state that allowed us to explore distinct chemical spaces around *Lc*-BPE and improve its affinity and selectivity for ASCT2 (e.g., ERA-9 and ERA-11).

In summary, this study described the mechanism of transport inhibition in an important therapeutic target, hASCT2, by combining modern methods in protein structure determination and computational modeling. Our structure of hASCT2 in complex with *Lc*-BPE accomplishes the following: 1) reveals a previously unknown substate with high affinity for potent inhibitors; 2) experimentally confirms which subpockets are targeted by the inhibitor; 3) allows the modeling of two distinct binding states, in which one was not previously predicted by homology modeling and ligand docking; 4) reveals an outward-open conformation potentially useful for drug design; and 5) combined with MD simulations, captures multiple ligand configurations that are highly relevant for pharmacological intervention. The relevance of this work is highlighted by the use of rational design to develop multiple inhibitors for ASCT2, which can be used to further characterize the role of hASCT2 in disease and as a framework for designing future clinically relevant compounds. Finally, the approach presented in this work is generally applicable to other challenging membrane protein targets such as the human SLC transporters.

## Methods

*SI Appendix* includes detailed information on the following: 1) molecular docking with Schrödinger; 2) relative binding affinity prediction; 3) MD simulations; 4) ASCT2 expression and purification; 5) reconstitution into proteoliposomes and transport assays; 6) reconstitution of ASCT2 in nanodiscs; 7) cryo-EM sample preparation and data collection; 8) image processing; 9) cryo-EM model building and validation; 10) cell culture and transfection; 11) cell viability assays; 12) electrophysiological techniques; 13) data analysis; 14) synthesis; and 15) NMR data for major intermediates.

## Supplementary Material

Supplementary File

## Data Availability

Our code is available on the A.S. laboratory GitHub repository: https://github.com/schlessinger-lab/. Details of the MD simulations as well as Protein Data Bank (PDB) files of representative conformations can be found on PLUMED-NEST: https://www.plumed-nest.org (accession code 20.015). The three-dimensional cryo-EM density maps of the outward-open ASCT2 in presence of *Lc*-BPE and the outward-open ASCT2 in presence of ERA-21 have been deposited in the Electron Microscopy Data Bank under accession nos. EMD-12142 and EMD-12143, respectively. The deposition includes the cryo-EM maps, both half-maps, the unmasked and unsharpened refined maps, and the mask used for final Fourier shell correlation (FSC) calculation. Coordinates of three models have been deposited in the PDB. The accession nos. for ASCT2 in the presence of *Lc*-BPE (position up) and ASCT2 in the presence of *Lc*-BPE (position down) are 7BCQ and 7BCS, respectively. All other study data are included in the article and/or *SI Appendix*.
