# Supplementary material for: Rational design of ASCT2 inhibitors using an integrated experimental-computational approach
Source: Proc Natl Acad Sci U S A. 2021 Sep 10;118(37):e2104093118. doi: 10.1073/pnas.2104093118 (PMC8449414; doi:10.1073/pnas.2104093118)
Supplement: Supplementary File [file pnas.2104093118.sapp.pdf]

Supplementary Information for

Rational design of ASCT2 inhibitors using an integrated experimental-computational approach

Rachel-Ann A. Garibsingh[1, †], Elias Ndaru[2, †], Alisa A. Garaeva[3, †], Yueyue Shi[2], Laura Zielewicz[2], Paul Zakrepine[2], Massimiliano Bonomi[4], Dirk J. Slotboom[3,6], Cristina Paulino[5]* ,Christof Grewer[2]*, Avner Schlessinger[1]*

*Corresponding authors. Email: c.paulino@rug.nl , cgrewer@binghamton.edu, avner.schlessinger@mssm.edu

This PDF file includes:

Supplementary Figures 1-11
Supplementary Table 1
Supplementary Methods:
1. Molecular docking with Schrödinger;
2. Relative binding affinity prediction;
3. MD simulations;
4. ASCT2 expression and purification;
5. Reconstitution into proteoliposomes and transport assays;
6. Reconstitution of ASCT2 in nanodiscs;
7. Cryo-EM sample preparation and data collection;
8. Image processing;
9. Cryo-EM model building and validation
10. Cell culture and transfection;
11. Cell viability assays;
12. Electrophysiological techniques;
13. Data analysis;
14. Synthesis;
15. NMR data for major intermediates
References 1- 3

Fig. S1

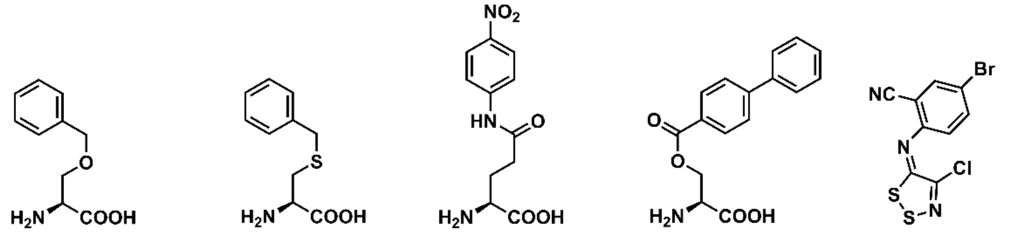

Grewer & Grabsch, 2004 (Ref. 2) Benzyserine **K_i 0.9 mM**

Grewer & Grabsch, 2004 (Ref. 2) Benzycysteine **K_i 0.78 mM**

Esslinger et al., 2004 (Ref. 9) GPNA **IC_{50} >1 mM**

Albers et al., 2011 (Ref. 1) compound 5c **K_i 30 μM**

Oppedinaso et al., 2012 (Reviewed in Ref. 21) compound 12 **IC_{50} >3.7 μM**

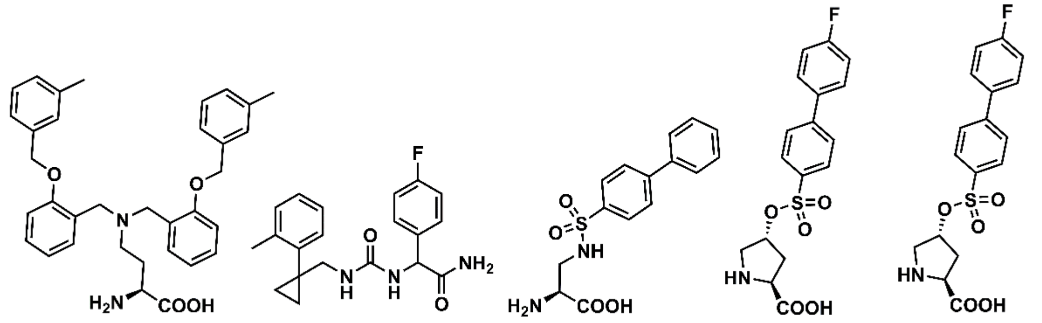

Schulte et al., 2018 (Reviewed in Ref. 21) V9302 **IC_{50} >9.6 μM**

Garibsingh et al., 2018 (Ref. 26) Compound 10 **IC_{50} >97 μM**

Ndaru et al., 2019 (Ref. 22) Compound 13b **K_i 410 μM**

Ndaru et al., 2019 (Ref. 22) Compound 15b **K_i 267 μM**

Ndaru et al., 2019 (Ref. 22) Compound 16b **K_i 8 μM**

Fig. S1. Selected published ASCT2 inhibitors.

Fig. S2

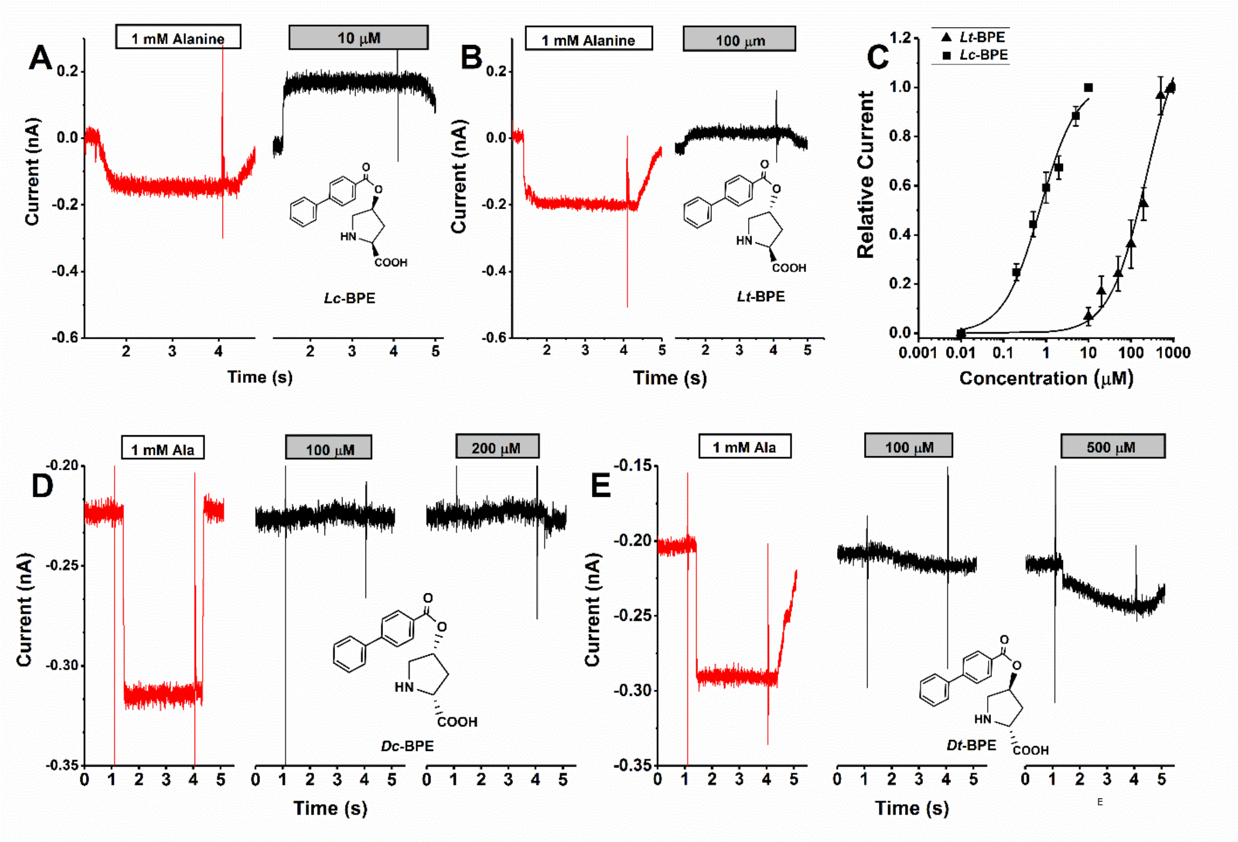

Fig. S2. Electrophysiological characterization of 4-hydroxyproline biphenyl ester diastereomers. (A & B) Original current traces of inward anion currents produced by the application of 1 mM alanine (red trace) and inhibition of leak anion current by the application of *Lc*-BPE (A) and *Lt*-BPE (B) to rASCT2 expressing cells. The white and grey bars illustrate the time of compound application to rASCT2. **(C)** Dose response curves for *Lc*-BPE (squares) and *Lt*-BPE (triangles). The solid lines represent fits according to Michaelis-Menten-like equation with Ki of 0.74 ± 0.11 µM and 232 ± 44 µM. **(D)** and **(E)** Analogous experiment as in **(A)** and **(B)** but D-isomers of 4-hydroxyproline, *Dc*-BPE **(D)** and *Dt*-BPE **(E)** were used. Currents from these two isomers were either too small at high compound concentrations (*Dc*-BPE) or unspecific (*Dt*-BPE) and therefore dose response curves and estimated Ki could not be obtained. Transmembrane potential was 0 mV in the presence of 130 mM NaSCN/10 mM Alanine internal, and 140 mM NaCl external.

Fig. S3.

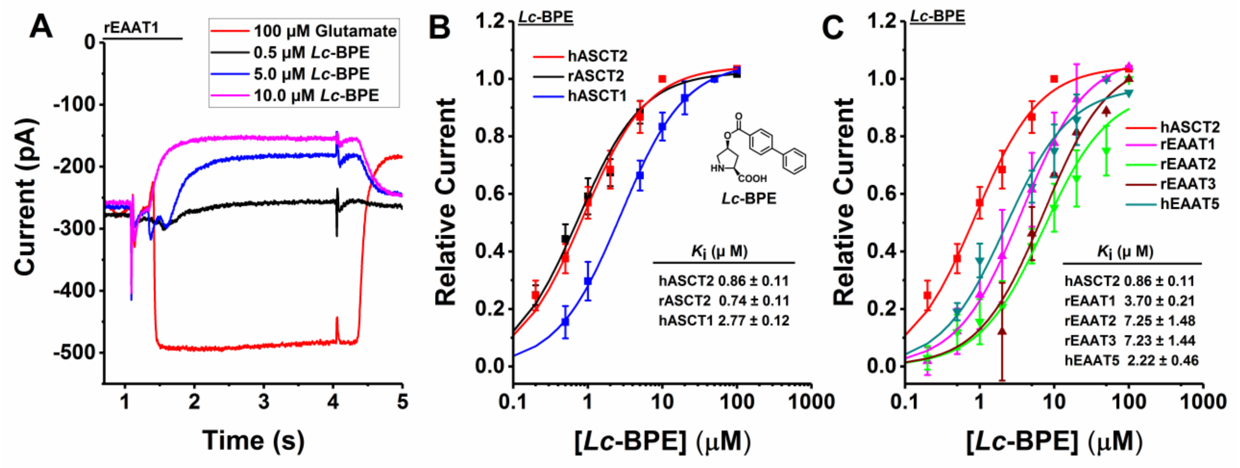

Fig. S3. SLC-1 specificity of *Lc*-BPE. (A) Electrophysiological current responses to the application of 100 µM glutamate (red trace) or varying concentrations of *Lc*-BPE (black, blue and red traces) to rEAAT1-expressing cells HEK293 cells. **(B-C)** Dose response curves obtained from currents recorded after application of *Lc*-BPE extracellularly to cells transiently expressing hASCT2, rASCT2, hASCT1 (panel B) and rEAAT1, rEAAT2, rEAAT3 and hEAAT5 (panel C) respectively. For hASCT2, rASCT2 and hASCT1, intracellular pipette solution contained 10 mM alanine and 130 mM NaSCN. For rEAAT1, rEAAT2, rEAAT3 and hEAAT5, intracellular solution contained 10 mM glutamate and 130 mM NaSCN. Extracellular solution was the same for all the experiments (140 mM NaCl). The voltage was 0 mV.

Fig. S4

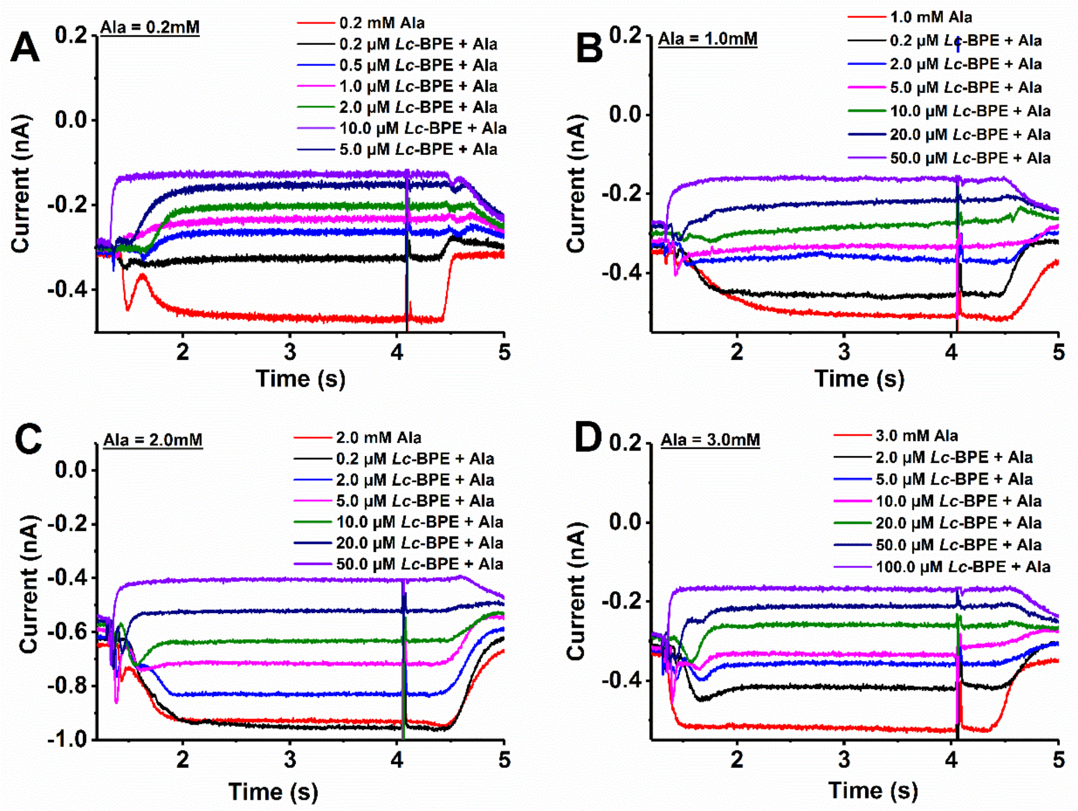

Fig. S4. *Lc*-BPE-induced currents in the presence of varying concentrations of alanine. Currents were produced by the application of different concentrations of alanine in the absence (red trace) and presence of varying *Lc*-BPE concentrations to HEK293T cells overexpressing rASCT2. (**A)** 0.2 mM alanine, **(B)** 1.0 mM Alanine, (**C)** 2.0 mM alanine and (**D)** 3.0 mM alanine. All experiments were done at 0 mV, intracellular and extracellular buffers consisted of 130 mM NaSCN and 140 mM NaCl respectively at pH 7.40. Both alanine and *Lc*-BPE were dissolved in extracellular buffer and applied externally.

Fig. S5

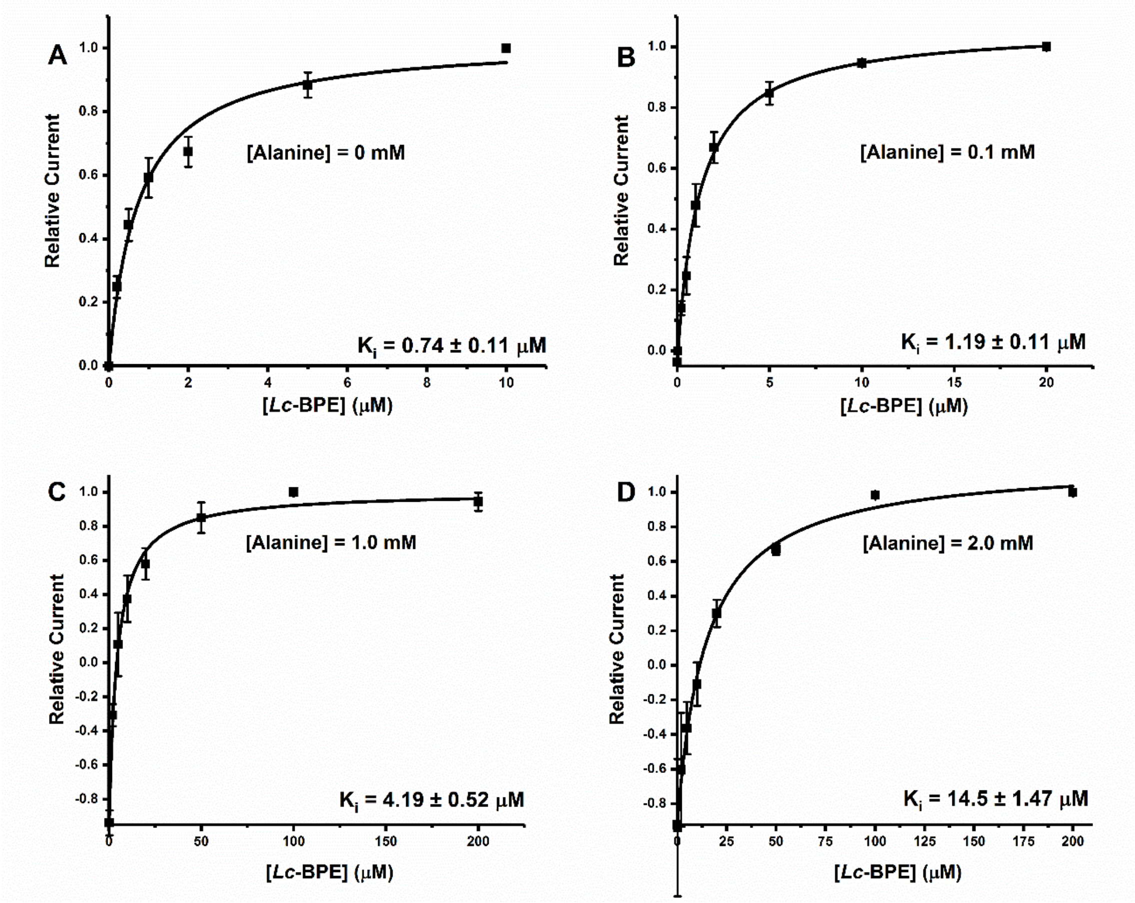

Fig. S5. *Lc*-BPE dose response relationships in the presence of varying concentrations of alanine. (A), no alanine, **(B)**, 0.1 mM alanine, **(C)**, 1.0 mM Alanine and **(D)**, 2.0 mM alanine. The lines represent fits of the currents according to the equation: $I = I1 + I2$ [Inh]/(Ki + [Inh]), where $I1$ is the alanine induced current without the inhibitor, and $I2$ is the maximum current in the presence of saturating inhibitor concentration, [Inh]max[1]. For **(A)** and **(B)**, currents are normalized to the current recorded after application of highest inhibitor concentration to rASCT2 expressing cells while **C** and **D** currents are normalized to the current recorded after application of alanine in the absence of inhibitor (those two approached results to the same Ki).

Fig. S6.

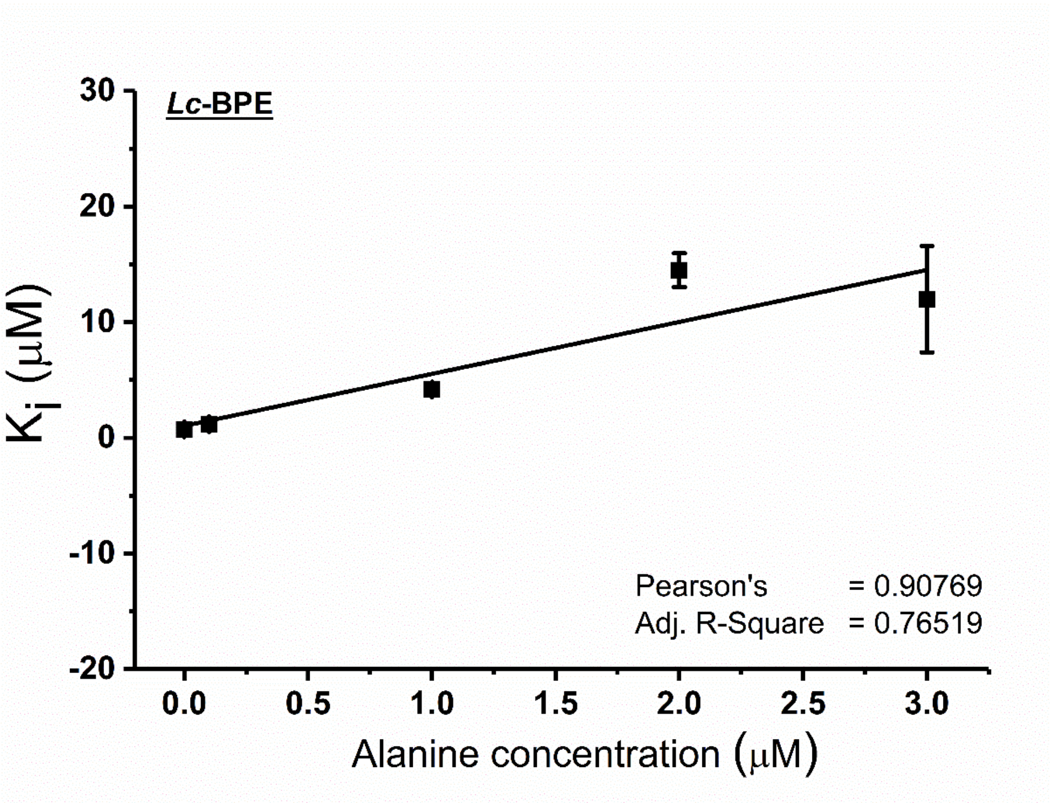

Fig. S6. *Lc*-BPE competes with substrate for the substrate binding site. *Lc*-BPE apparent affinity (Ki) (obtained from Fig. S 6) plotted as a function of alanine concentration, exhibiting a linear relationship according to the equation Ki(Ala) = Ki(0) + [Ala]Ki(0)/Km(Alanine), Ki(Ala) and Ki(0) are Ki values in the presence and absence of alanine while Km(Alanine) is the apparent Michaelis-Menten constant for alanine activation of inwardly directed anion current[2]. Pearson's r value is 0.91 and adjusted R^2 is 0.77 which demonstrates good correlation.

Fig. S7

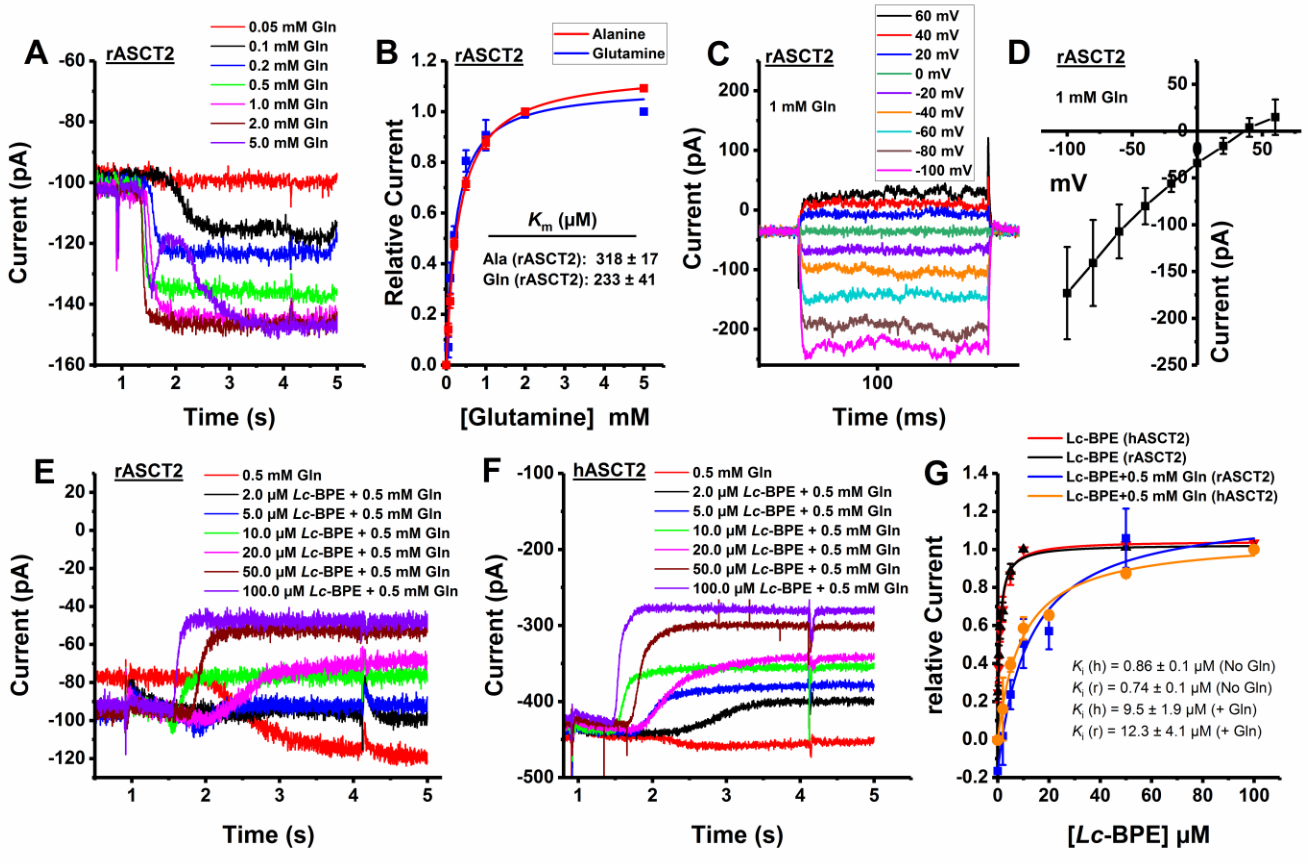

Fig. S7. *Lc*-BPE-induced currents in the presence of glutamine. (A) Current traces obtained when varying concentrations of glutamine are applied to rat ASCT2-expressing HEK293 cells. **(B)** Dose response relationships for alanine and glutamine with a rat ASCT2 apparent K_m of 318 ± 17 µM and 233 ± 41 µM respectively. **(C)** Current traces obtained when 1 mM glutamine was applied to rat ASCT2 expressing cells and cell membrane potential rapidly changed from +60mV to -100 mV. **(D)** Current-Voltage (I-V) curves obtained from experiment in C. Background currents were subtracted. **(E-F)** Current traces from increasing concentrations of *Lc*-BPE in presence of 0.5 mM glutamine for rASCT2 and hASCT2 respectively. **(G)** Dose response relationships for *Lc*-BPE in absence of glutamine (hASCT2 (red), rASCT2 (black)) and presence of 0.5 mM glutamine (hASCT2 (orange), rASCT2 (blue). All experiments were done at 0 mV, intracellular and extracellular buffers consisted of 130 mM NaSCN and 140 mM NaCl respectively at pH 7.40. Both glutamine and *Lc*-BPE were dissolved in extracellular buffer and applied externally.

Fig. S8.

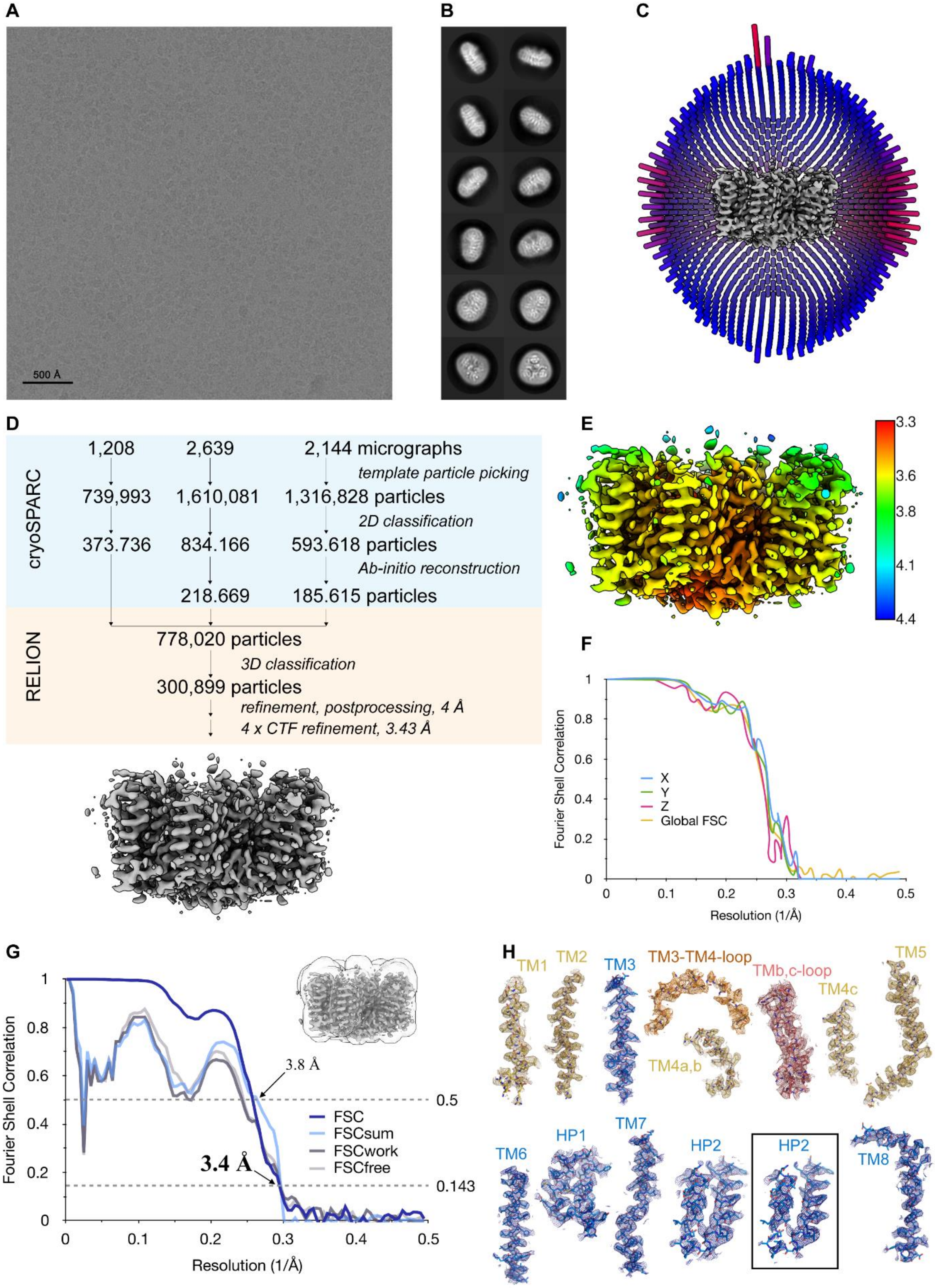

A
500 Å
B
C
D
cryoSPARC
1,208
2,639
2,144 micrographs
template particle picking
739,993
1,610,081
1,316,828 particles
2D classification
373.736
834.166
593.618 particles
Ab-initio reconstruction
218.669
185.615 particles
RELION
778,020 particles
3D classification
300,899 particles
refinement, postprocessing, 4 Å
4 x CTF refinement, 3.43 Å
E
3.3
3.6
3.8
4.1
4.4
F
Fourier Shell Correlation
X
Y
Z
Global FSC
Resolution (1/Å)
G
Fourier Shell Correlation
3.8 Å
3.4 Å
0.5
0.143
FSC
FSCsum
FSCwork
FSCfree
Resolution (1/Å)
H
TM1
TM2
TM3
TM3-TM4-loop
TMb,c-loop
TM4c
TM5
TM4a,b
TM6
HP1
TM7
HP2
HP2
TM8

Fig. S8. Cryo-EM reconstruction of ASCT2 nanodiscs in the presence of inhibitor Lc-BPE. Representative cryo-EM image **(A)** and 2D-class averages **(B)** of vitrified ASCT2 nanodiscs in the presence of inhibitor. **(C)** Angular distribution plot of particles included in the final C3-symmetrized 3D reconstruction. **(D)** Image processing workflow. **(E)** Final reconstructed map colored by local resolution, as estimated in Relion. **(F)** Anisotropy estimation plot of the final map. The global FSC curve is represented in yellow. The directional FSCs along the x, y and z axis are displayed in blue, green and red, respectively. **(G)** FSC plot used for resolution estimation and model validation. The gold-standard FSC plot between two separately refined half-maps is shown in blue and indicates a final resolution of 3.4 Å. The FSC model validation curves for FSCsum, FSCwork and FSCfree, as described in material and methods, are shown in light blue, dark grey and light grey respectively. A thumbnail of the mask used for FSC calculation overlaid on the map is shown in the upper right corner. Dashed lines indicate the FSC thresholds used for FSC of 0.143 and for FSCsum of 0.5. **(H)** Cryo-EM densities. Shown are selections of cryo-EM densities of ASCT2 nanodisc in presence of inhibitor, with the respective refined models superimposed. Models are shown as sticks and structural elements are labelled. Transmembrane helices (TM) of the transport domain are coloured in blue, of the scaffold domain in yellow, the loop between $TM4_b$ and $TM4_c$ in red, the loop between TM3 and TM4 in orange. Densities were sharpened with a b-factor of -189 Å2. Densities of TM1, TM6, TM7 and HP2 were contoured at 3 σ; TM2, TM3, TM4a,b, TM4c, TM5, TM8, HP1 were contoured at 4 σ; $TM4_{b,c}$ loop, TM3-TM4 loop was contoured at 2 σ. To highlight the poorly resolved density for HP2 which is indicative of a higher flexibility the same region is countered at 4 σ and marked by a black rectangle.

Fig. S9.

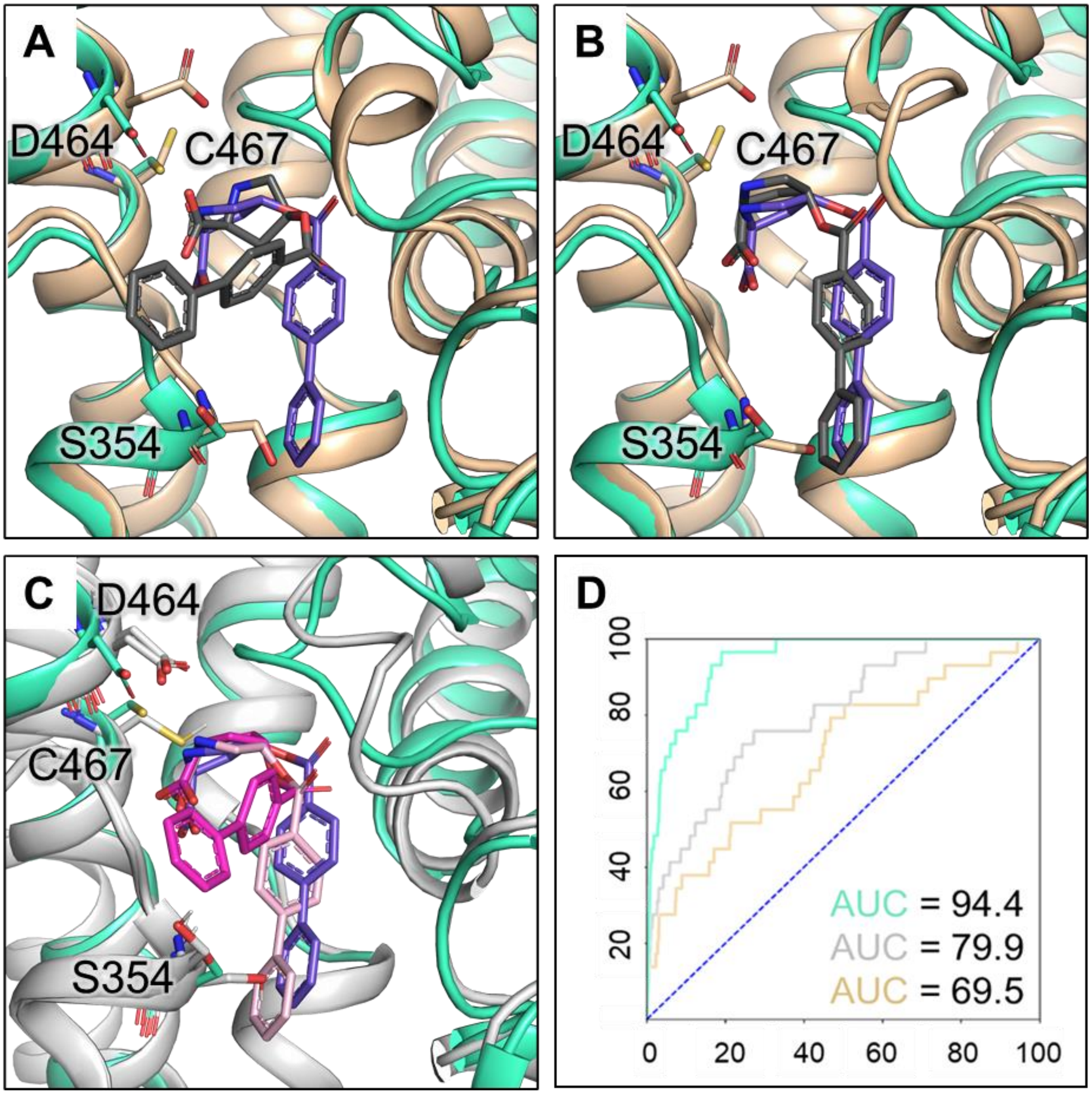

Fig. S9. Progressive model building and refinement of the binding site. **(A** and **B)** Superposition of the homology model (green cartoon with purple *Lc*-BPE) and initial cryo-EM structures (tan cartoon with dark gray *Lc*-BPE) with **(A)** "ligand up" and **(B)** "ligand down" conformations; showing the steric clash between the S354 sidechain and *Lc*-BPE. **(C)** Superposition of refined "ligand up" and "down" structures with the homology model. **(D)** Enrichment plots of ASCT2 homology model (green) and initial "ligand down" structure (tan) and after sidechain refinement (gray). The plot for a random selection of ligands is represented by the blue dashed line.

Fig. S10.

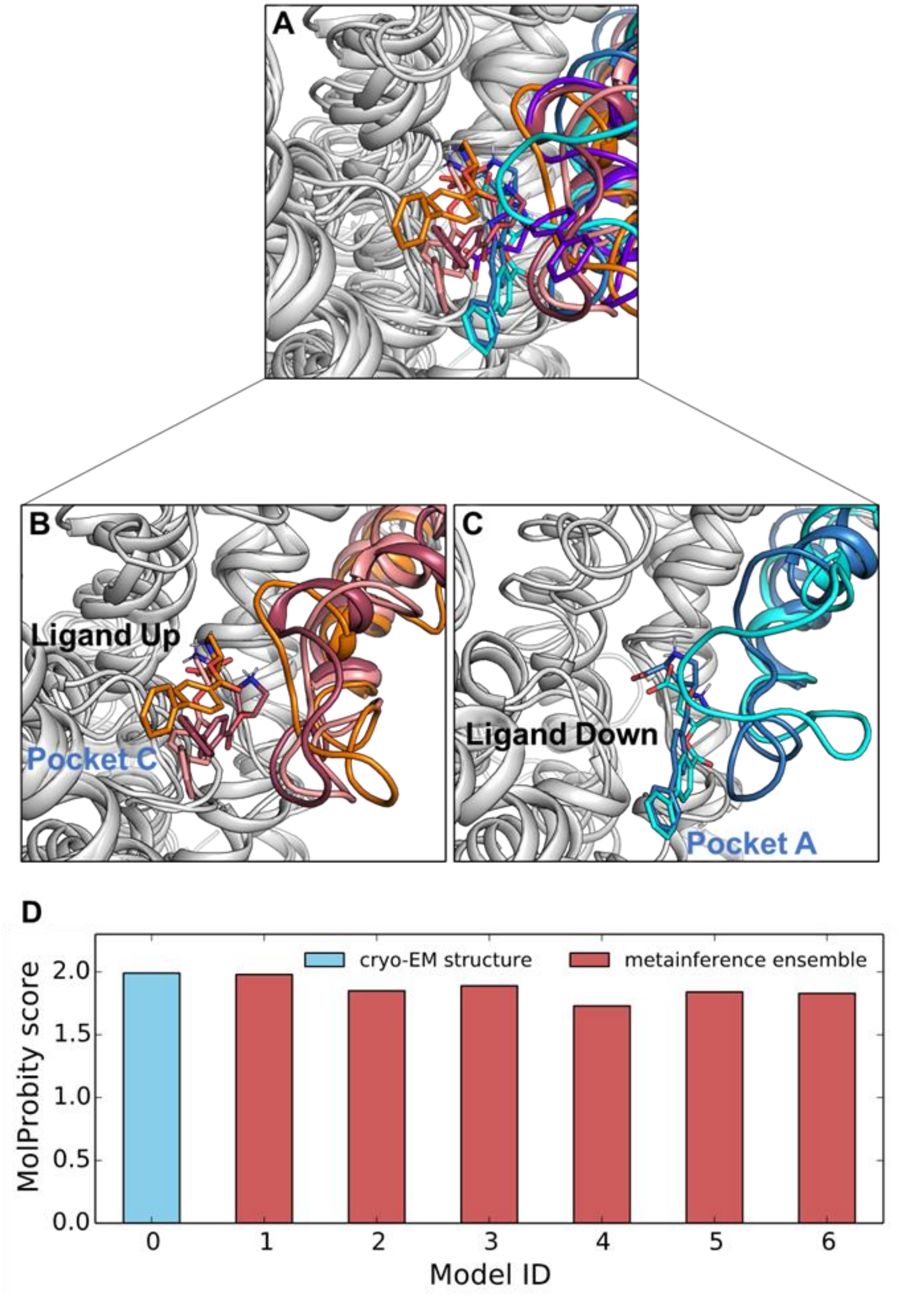

Fig. S10. Clusters of ligand conformations obtained with metainference molecular dynamics (MD) simulations. (A) Superposition of 6 clusters of ligand conformations in the substrate binding site. **(B)** “Ligand up” clusters in red (69%), salmon (7%) and orange (6%). **(C)** “Ligand down” clusters in dark blue and cyan represent 13% of all clusters. The purple cluster represents an outlier cluster that is outside of the binding site and is unlikely to be physiological. **(D)** Stereochemical quality of the cryo-EM structure and metainference ensemble. We assessed the stereochemical quality of the

cryo-EM structure (cyan, model 0) as well as of the six representative clusters from the metainference ensemble (red, models 1-6) using the MolProbity score[3]. This score provides a global measure of the quality of the models in terms of number of steric clashes, rotamer outliers, and percentage of backbone Ramachandran conformations outside favored regions. The lower the score, the better the quality of the models.

Fig. S11

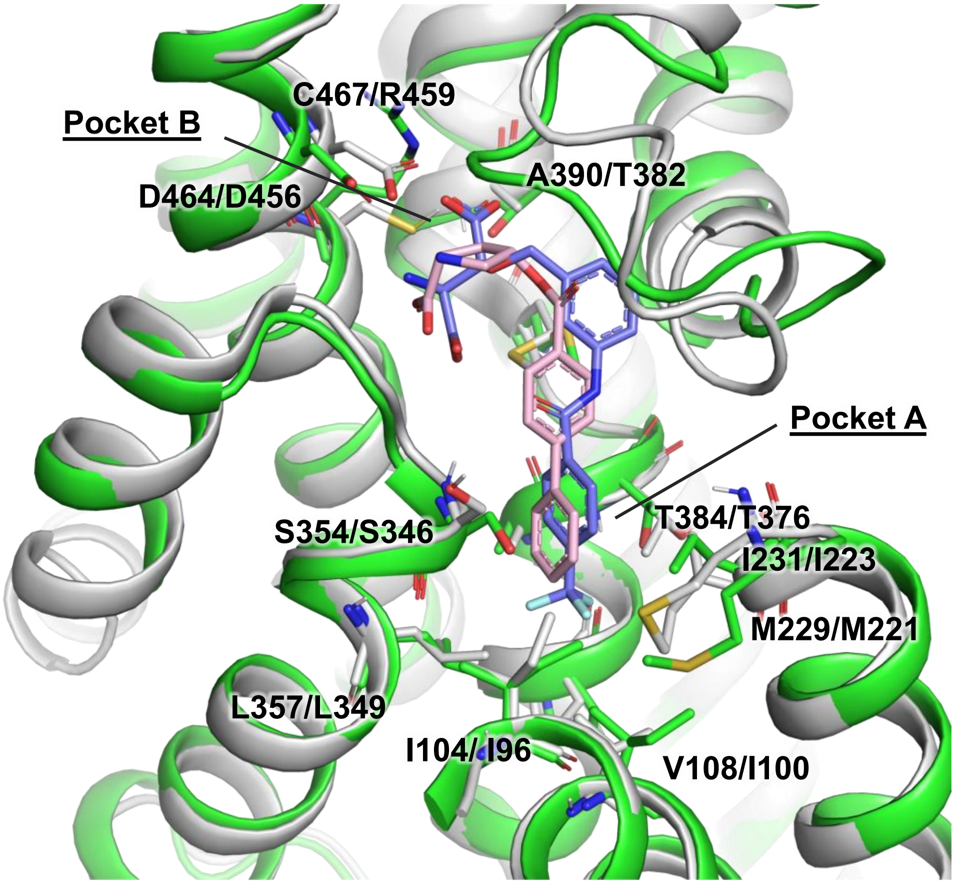

Fig. S11. Superposition of ASCT2 "ligand down" and EAAT1 (PDB ID: 5MJU) structures. ASCT2 is shown in gray cartoon with *Lc*-BPE represented by pink sticks. EAAT1 is shown in green cartoon with the EAAT1 inhibitor TFB-TBOA represented by purple sticks. Key residues of the binding site are highlighted with sticks and labeled with the residue numbers of ASCT2 followed by EAAT1. The conserved Pocket A and variable Pocket B are also labeled.

SI Table 1: Second generation inhibitors

Name	Structure	Binding affinity K_i (μM)	Docking score	Docking pose	MM-GBSA
ERA-4	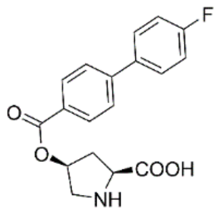	hASCT2 0.74 ± 0.09<br>rASCT2 0.65 ± 0.04<br>hASCT1 0.90 ± 0.08<br>rEAAT1 0.40 ± 0.07<br>rEAAT2 1.29 ± 0.31<br>rEAAT3 0.69 ± 0.07<br>hEAAT5 2.40 ± 0.72	-6.51 †	ligand down	-40.42
ERA-5	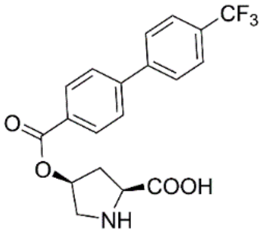	hASCT2 1.05 ± 0.21<br>rASCT2 0.27 ± 0.05<br>hASCT1 0.44 ± 0.14<br>rEAAT1 1.00 ± 0.25<br>rEAAT2 0.97 ± 0.10<br>rEAAT3 1.25 ± 0.24<br>hEAAT5 1.05 ± 0.15	-6.46 †	ligand down	-30.03
ERA-6	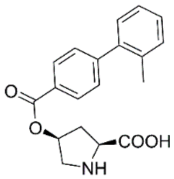	hASCT2 1.12 ± 0.11<br>rASCT2 0.97 ± 0.08<br>hASCT1 1.33 ± 0.30<br>rEAAT1 1.00 ± 0.11<br>rEAAT2 1.30 ± 0.13<br>rEAAT3 1.99 ± 0.36<br>hEAAT5 2.50 ± 0.93	-5.86 †	ligand down	-46.6
ERA-11	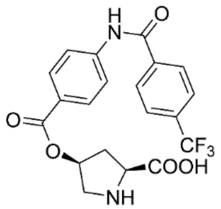	hASCT2 2.94 ± 0.93<br>rASCT2 3.37±0.41<br>hASCT1 8.51±3.87**<br>rEAAT1 9.6 ± 7.5<br>rEAAT2 No current §<br>rEAAT3 No current §<br>hEAAT5 No current §	-5.05 †	other	-36.7
ERA-16	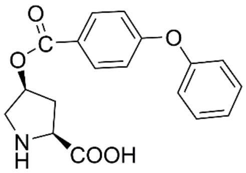	hASCT2 4.17 ± 0.46<br>rASCT2 3.68±0.73<br>hASCT1 5.71±0.91<br>rEAAT1 8.18±1.77<br>rEAAT2 7.72±1.89<br>rEAAT3 10.27±2.15<br>hEAAT5 6.17±1.47	-5.78 †	ligand down	-46.1

Name	Structure	Binding affinity K_i (μM)	Docking score	Docking pose	MM-GBSA
ERA-9	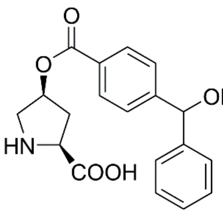	hASCT2 5.65 ± 1.30<br>rASCT2 18.80 ± 4.97<br>hASCT1 43.00 ± 13.41<br>rEAAT1 39.46 ± 19.10<br>rEAAT2 No current ‖<br>rEAAT3 No current ‖<br>hEAAT5 No current ‖	-6.35 †	ligand down	-37.3
ERA-8	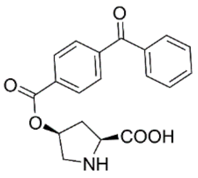	hASCT2 21.39 ± 3.21<br>rASCT2 6.66 ± 1.17<br>hASCT1 6.86 ± 1.14<br>rEAAT1 21.20 ± 4.46<br>rEAAT2 15.40 ± 4.87<br>rEAAT3 No current ‖**<br>hEAAT5 13.81 ± 2.67<br>*****hASCT2 **IC_{50}** 207	-5.77 †	ligand down	-43.52
ERA-21	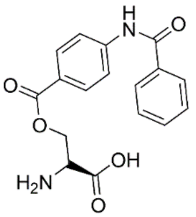	hASCT2 22.63 ± 3.66<br>rASCT2 4.33 ± 1.30<br>hASCT1 11.64 ± 1.75<br>rEAAT1 9.57 ± 1.61<br>rEAAT2 11.01 ± 1.25<br>rEAAT3 12.26 ± 1.86<br>hEAAT5 21.26 ± 3.15<br>*****hASCT2 **IC_{50}** 56	-5.21 †	ligand down	-43.33
ERA-25	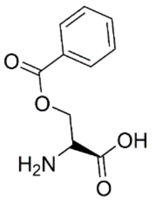	hASCT2 185.8 ± 17.2<br>rASCT2 290.3 ± 16.5<br>hASCT1 169.6 ± 26.7<br>rEAAT1 195.1 ± 48.4<br>rEAAT2 163.8 ± 47.0<br>rEAAT3 656.0 ± 115<br>hEAAT5 No current #	-4.90 ‡	other	-25.9
ERA-28	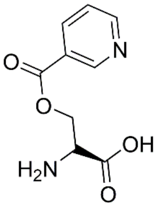	hASCT2 690.0 ± 54.2<br>rASCT2 265.7± 42.7<br>hASCT1 548.8 ± 306<br>rEAAT1 No current #<br>rEAAT2 No current #<br>rEAAT3 No current #<br>hEAAT5 No current #	-4.87 ‡	other	-23.25
ERA-35	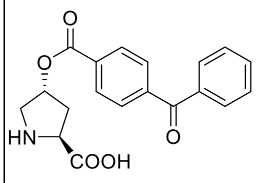	hASCT2 >500<br>rASCT2 120.8 ± 29.9<br>hASCT1 No Data<br>rEAAT1 No current #<br>rEAAT2 No current #<br>rEAAT3 No current #	-4.91 †	other	-33.78

Name	Structure	Binding affinity K_i (µM)	Docking score	Docking pose	MM-GBSA
		hEAAT5 No current [#]<br>***hASCT2** IC_{50} 411			
ERA-31	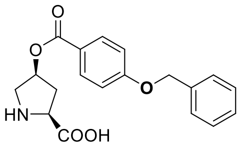	hASCT2 No current [||**]<br>rASCT2 8.17±1.05<br>hASCT1 >100<br>rEAAT1 No current [||]<br>rEAAT2 No current [||]<br>rEAAT3 No current [||]<br>hEAAT5 No current [||]	-5.71 [†]	ligand down	-38.4
ERA-3	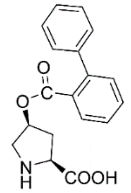	hASCT2 No current [§]<br>rASCT2 No current [||**]<br>hASCT1 No current [||**]<br>rEAAT1 No current [||]<br>rEAAT2 No current [||]<br>rEAAT3 No current [||]<br>hEAAT5 No current [||]	-6.18 [†]	other	-44.79
ERA-29	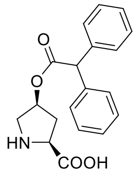	hASCT2 No current [||]<br>rASCT2 No current<br>hASCT1 No Data<br>rEAAT1 No current [§]<br>rEAAT2 No current [§]<br>rEAAT3 No current [§]<br>hEAAT5 No current [§]	-5.11 [‡]	Other	-59.05

Name marks the compound name.
Structure corresponds to the 2D structure of the compound drawn by PerkinElmer ChemDraw
Binding affinity marks the experimentally Ki values
Docking score marks the docking score of the top scoring pose using Glide
MM-GBSA corresponds to the predicted free energy of binding and corresponds with the docking parameters used and specified in the docking score column.
Docking pose represents whether the ligand occupies a pose similar to the inhibitor in the “ligand up” or “ligand down” structures. Poses labeled “other” means the ligand pose was poor and did not reflect either of the “ligand up” or “ligand down” conformations.
* IC50 (µM) measured in transport assay in proteoliposomes in the presence of 5 µM glutamine
† Molecular docking to “ligand down” structure without constraints.
‡ Molecular docking to “ligand up” structure without constraints.
"No current" indicates that no outward current (i.e., inhibition of anion leak current) was observed in these experiments. This means that the compound either does not bind or binds but is unable to block the leak anion current. Superscripts §, ||, ¶ and #indicate the highest inhibitor concentration tested in µM where; 20 ≤ § ≤ 50, 100 ≤ || ≤ 400, 500 ≤ ¶ ≤ 1000 and 1500 ≤ # ≤ 2500. Asterisk (**) indicates that an extra experiment (s) was performed in the presence of substrate (alanine or glutamate).

Supplementary Materials and Methods

We have previously developed homology models of human ASCT2 (hASCT2) based on the outward-open structures of the human EAAT1 transporter (EAAT1; 46% sequence identity) [4,5]. Here, we iteratively refined this model based on its ability to discriminate ligands from decoy compounds with ligand enrichment calculations. The sidechains of the binding site residues S354, D464, and C467 were remodeled on fixed backbone using PyMOL[6] and SCRWL4[7], guided by recently determined structures of hASCT2 in multiple conformations. PyMOL mutagenesis was done with backbone dependent rotamers and SCRWL4 was done according to default parameters. Enrichment was done using a library of 29 known ASCT2 ligands, including substrates and inhibitors that were collected from the literature[1,8-12] and ChEMBL[13] and 1,434 decoys generated with the DUD-E server[14]. Docking was performed with OpenEye FRED[15] as described in our previous work[5]. Hydrogen-bond constraints used for docking against the model at HP1; S351 and S353, and the refined structure at TM8; D464 and TM8; N471.

Molecular docking with Schrödinger

All docking calculations were performed with the Schrödinger suite using Glide v19-3[16]. In brief, for the initial compound series (Table 1) the models were prepared for docking using the Maestro Protein Preparation Wizard under default parameters. The substrate binding site was defined using the Maestro Receptor Grid Generation panel and the coordinates of the reference ligand (TFB-TBOA) were derived from the EAAT1 template structure. The allosteric ligand UCPH101 was removed from the model prior to defining the site, to avoid multiple reference ligands in the grid. Docking was conducted without constraints. Compounds were prepared for docking with Glide v19-3 with LigPrep under default parameters[16].

Compounds for the 2nd compound series (Table 3) were docked in the unrefined and refined “ligand up” and “ligand down” cryo-EM structures, with and without constraints using Glide v19-3. Both protein and ligand preparation and was carried out as described above, and grid generation was performed with the Maestro Receptor Grid Generation panel using the coordinates of the reference ligand (*Lc-BPE*) from the cryo-EM structure. In general, two the “best” overall docking scores and poses came from refined structures without constraints. (Table 3).

Relative binding affinity prediction

We estimated the relative binding affinity between the compounds and the ASCT2 model binding site with mechanics generalized with born surface area solvation (MM-GBSA). These calculations were relative, where a more negative value was indicative of a better binding affinity. Here we used MM-GBSA with Prime from the Schrödinger suite v18-4)[16]. The model was prepared as described above with the exception that reference ligands were removed prior, and we used the docked ligands from above as input. Standard parameters were used, with the distance from the ligand set to 5 Å for all calculations. For the second compound series, the best docking poses and scores for the “ligand up” and “down” structures were calculated using docking from the refined structures without constraints.

MD simulations

The atoms of chain A and of the associated ligand were extracted from the ‘ligand up’ cryo-EM structure. CHIMERA[17] was used to select the cryo-EM density within 5 Å of the model to be used as input of the metainference simulation of the ASCT2 monomer. The starting model was prepared using CHARMM-GUI[18]. 113 POPC lipids were added to the system along with 11554 water molecules in a triclinic periodic box of volume equal to 616 nm^3. 31 potassium and 30 chloride ions were added to ensure charge neutrality and a salt concentration of 0.15 M. The CHARMM36 force field[19] was used for the protein, lipids, and ions; the CHARMM General Force Field and the TIP3P model were used for the ligand and water molecules, respectively. A 30 ns-long equilibration was performed following the standard CHARMM-GUI protocol consisting in multiple consecutive simulations in the NVT and NPT ensembles. During these equilibration steps, harmonic restraints on the positions of the lipids, ligand, and protein atoms were gradually switched off.

In the metainference simulation, a Gaussian noise model with one error parameter for each voxel of the cryo-EM map was used. These variables were marginalized to avoid their explicit sampling, as done in previous applications[20,21]. 16 replicas of the system were used, and their initial configurations were randomly selected from the last 10ns-long step of the equilibration protocol. The metainference run was conducted for a total aggregated time of 1.8 μs. All simulations were carried out using GROMACS 2019.6 [22] and the ISDB module[23] of the open-source, community-developed PLUMED[24] library (GitHub ISDB branch; https://github.com/plumed/plumed2/tree/isdb). For the analysis, the initial frames of the trajectory of each replica, corresponding to 20% of the total simulation time, were considered as additional equilibration steps under the cryo-EM restraint and discarded. The remaining conformations from all replicas were merged together and clustered using: i) the Root Mean Square Deviation calculated on all the heavy atoms of the ligand and of the protein residues within 5 Å of the ligand in at least one member of the ensemble; ii) the gromos algorithm[25] with a cutoff equal to 2.5 Å. PDB of the starting model, topology files, GROMACS inputs for equilibration and production runs, PLUMED input files for the metainference simulation, and analysis scripts are available on PLUMED-NEST[24] (www.plumed-nest.org) under accession code plumID:20.015.

Cell culture and transfection

Human embryonic kidney 293 (HEK293, ATCC CRL-11268) cells were cultured in DMEM media supplemented with 10% (*v/v*) fetal bovine serum (FBS), 2 mM glutamine, 1% penicillin streptomycin solution, 1 mM sodium pyruvate and non-essential amino acids. Cells were maintained at 37^0C in a fully humidified atmosphere containing 5% CO2. rASCT2, hASCT2, hASCT1, EAAT1, EAAT2, EAAT3, EAAT5 and YFP complementary DNAs were each used to transiently transfect HEK293 using POLYPLUS Jet-prime transfection reagent. Cells were analyzed using electrophysiological techniques 24-40 hours after transfection.

Electrophysiological techniques

Electrophysiological experiments were performed as described previously[1,2]. Stock solutions of inhibitor was prepared in dimethyl sulfoxide (DMSO) up to 100 mM.

Dilutions to working concentrations were made using external buffer. The highest DMSO concentration used (2%) did not affect electrophysiological results in control cells. For rASCT2, hASCT2 and hASCT1, external buffer contained 140 mM NaCl, 2 mM MgCl2, 2 mM CaCl2, and 10 mM HEPES, pH 7.40 while internal pipette solution comprised of 130 mM NaSCN, 2 mM MgCl2, 10 mM EGTA, 10 mM HEPES and 10mM alanine, pH 7.40. For specificity experiments with EAAT1, EAAT2, EAAC1 and EAAT5, internal solution contained 10 mM glutamate instead of alanine. Compounds were applied to HEK293 cells expressing DNA of interest suspended from a current recording electrode in whole cell configuration[44] through a rapid solution exchange device described previously[45]. Cells are immersed in external buffer bath used to dissolve the inhibitors. The open pipette resistance was between 3 and 6 MΩ. Series resistance was not compensated in these experiments due to relatively small currents. Currents traces were recorded using an Adams and List EPC7 amplifier and digitized using a Molecular Devices Digidata A/D converter.

Data analysis

Data analysis was performed as described previously[46]. Linear and nonlinear curve fitting of the experimental data were analyzed using MicroCal Origin software. Linear plots were fitted using the general equation (y = a + bx) obtaining adjusted R^2 and Pearson's r values. Nonlinear dose–response relationships were fitted with a Michaelis–Menten-like equation to obtain apparent Ki values in the absence of substrate. For competition studies, equation $I = I1 + I2$ [Inh]/(Ki + [Inh]) was used for fitting, where *I*1 is the alanine induced current without the inhibitor, and *I*2 is the maximum current in the presence of saturating inhibitor concentration, [Inh]max[1,2]. At least five experiments were performed for each compound. Unless stated otherwise, the error bars in all our graphs represent mean ± SD**.**

ASCT2 expression and purification

Human ASCT2 (hASCT2) was produced in *Pichia pastoris* X-33 strain (Invitrogen) in fermentor[26] and purified using DDM and CHS (Anatrace) and 1 mM L-glutamine (Merck) to maintain protein stability [27]. Membranes representing ~1.5 g cells were solubilized in buffer A (25 mM Tris-HCl, pH 7.4, 300 mM NaCl, 10% (vol/vol) glycerol, 1 mM L-glutamine, 1% DDM and 0.1% CHS) for 1 h at 4 °C; ultracentrifuged (30 min, 442,907 × g, 4 °C) and supernatant containing solubilized protein was incubated with Ni2+-Sepharose resin for 1 h at 4 °C. Protein was eluted with buffer B (20 mM Tris-HCl, pH 7.4, 300 mM NaCl, 500 mM imidazole, pH 7.4, 10% glycerol, 1 mM L-glutamine, 0.02% DDM and 0.002% CHS), and applied to size-exclusion chromatography with a Superdex 200 10/300 gel-filtration column (GE Healthcare) preequilibrated with buffer C (20 mM Tris-HCl, pH 7.4, 300 mM NaCl, 1 mM L-glutamine, 0.02% DDM and 0.002% CHS). Peak elution fractions were immediately used in further procedures.

Reconstitution into proteoliposomes and transport assays

Freshly purified ASCT2 was reconstituted in the liposomes composed of *Escherichia coli* polar lipids and egg phosphatidylcholine at a 3:1 ratio (w/w) and supplemented with 10% (w/w) cholesterol (Avanti Polar Lipids)[27]. For transport assays proteoliposomes were loaded with 50 mM NaCl and 10 mM or 5 mM glutamine using three freeze-

thawing cycles, then extruded 11 times through a 400-nm-diameter polycarbonate filter (Avestin), diluted in buffer D (20 mM Tris pH 7.0) and collected during ultracentrifugation (45 min, 442,907 × g, 4 °C). Proteoliposomes were resuspended in buffer D (~1 µg protein per 1.5 µl) and used in the transport assays carried out in a water bath at 25 °C with constant stirring. Transport was initiated by dilution of 1.5 µl proteoliposomes in 80 µl external buffer (50 mM NaCl and 50 µM or 5 µM [3H]glutamine (PerkinElmer) in 20 mM Tris pH 7.0). Inhibitors or equivalent amounts of DMSO were added to the external buffer mixture. At indicated time points the reaction was stopped by diluting the mixture in 2 ml of cold buffer D, filtered over a 0.45-µm pore-size filter (Portran BA-85, Whatman), washed with 2 ml of cold buffer D and filtered again. The level of radioactivity accumulated inside the proteoliposomes, as a consequence of amino-acid exchange, was counted using a PerkinElmer Tri-Carb 2800RT liquid scintillation counter after dissolving the filter in 2 ml of scintillation liquid (Emulsifier Scintillator Plus, PerkinElmer).

Reconstitution of ASCT2 in nanodiscs

An aliquot of mixture of *E.coli* polar lipids and egg phosphatidylcholine (3:1, w/w) supplemented with 10% (w/w) cholesterol was solubilized with 30 mM DDM-CHS for 3 h while nutating. Nanodiscs were assembled at a reconstitution ratio of 1 nmol ASCT2 (as monomer): 5 nmol of MSP2N2: 70 nmol of solubilized lipids. For this freshly purified ASCT2 was first mixed with solubilized lipids and incubated for 30 min at 4 °C. Purified and TEV protease treated MSP2N2[28] was added for the following 30 min. 300 mg of SM2 BioBead (Bio-Rad) were added overnight to remove DDM. Assembled nanodiscs were purified from discs devoid of ASCT2 via Ni^{2+}-Sepharose chromatography, collected in elution fraction in buffer E (20 mM Tris-HCl, pH 7.4, 300 mM NaCl, 500 mM imidazole, pH 7.4) and further applied on size-exclusion chromatography with a Superdex 200 10/300 gel-filtration column preequilibrated with buffer F (20 mM Tris-HCl, pH 7.4, 200 mM NaCl).

Cryo-EM sample preparation and data collection

Freshly purified hASCT2 nanodiscs were concentrated to ~1 mg ml−1 using an Amicon Ultra-0.5 mL concentrating device (Merck) with a 100 kDa filter cut-off and then 100 μM inhibitor was added and incubated for 1 h on ice. 2.8 μl of the sample were applied onto the holey-carbon cryo-EM grids (Au R1.2/1.3, 300 mesh, Quantifoil), which were preliminary glow discharged at 5 mA for 30 s, blotted for 3–4 s in a Vitrobot Mark IV (Thermo Fisher) at 15 °C and 100% humidity, plunge frozen into a liquid ethane/propane mixture and stored in liquid nitrogen until further use. Screening of the grid areas with best ice properties was done with the help of a self-written script to calculate the ice thickness[29]. Cryo-EM data in selected grid regions were collected in-house on a 200-keV Talos Arctica microscope (Thermo Fisher) with a post-column energy filter (Gatan) in zero-loss mode, with a 20-eV slit and a 100-μm objective aperture. Images were acquired in an automatic manner with EPU (Thermo Fisher) and SerialEM on a K2 summit detector (Gatan) in counting mode at ×49,407 magnification (1.012 Å pixel size) and a defocus range from −0.9 to −1.9 μm. During an exposure time of 9 s, 60 frames were recorded with a total dose of about 53 electrons/Å2. On-the-fly data quality was monitored using FOCUS software[30].

Image processing

For the ASCT2 nanodiscs dataset in the presence of inhibitor *Lc*-BPE, 6,233 micrographs were recorded. Beam-induced motion was corrected with MotionCor2_1.2.1[31] and the CTF parameters estimated with ctffind4.1.13[32]. Recorded micrographs were manually checked in FOCUS (1.1.0), and micrographs, which were out of defocus range (<0.4 and >2 μm), contaminated with ice or aggregates, and with a low-resolution estimation of the CTF fit (>4 Å), were discarded. The remaining 5,991 micrographs were imported in cryoSPARC v2.14.2[33]. Around 1000 particles were manually picked to create templates for particle autopicking. 3,666,842 particles were autopicked and extracted with a box size of 200 pixels. After 2D classification 1,801,520 particles were left and majority of particles (1,427,784) were used for several rounds of ab-initio volume generation, and C3 symmetry was applied. 404,284 particles of best classes and 373,736 particles remaining after 2D classification (778,020 particles in total) were exported from cryoSPARC and imported in RELION-3.0.8[34] and used in 3D classification with C3 symmetry applied and resulted in a one best class with 300,899 particles (38.7%). These particles were used in the refinement job, where hASCT2 map generated in cryoSPARC was used as a reference and was low-pass filtered to 15 Å, and C3 symmetry was applied. In the last refinement iteration, a mask excluding nanodisc was used and the refinement continued until convergence (focus refinement), following postprocessing job, which resulted in a map at 4 Å. Four rounds of per-particle CTF refinement and beam tilt refinement in Relion3[8] improved resolution to 3.43 Å.

A similar approach was performed for the image processing of the ASCT2 nanodiscs dataset in the presence of ERA-21, In short, 9,788 micrographs were recorded, and 7,934 used for image processing after selection. 4,438,395 particles were autopicked and subjected to 2D classification in cryoSPARC. 1,308,359 selected particles were imported in RELION 3.1.0, refined and subjected to several rounds of per-particle CTF refinement. These particles with corrected defocus values were used for subsequent 3D

classification with C3 symmetry applied, after which 363,193 particles (28%) were refined, subjected to 2 rounds of per-particle CTF refinement and used in 3D classification. The best class included 224,884 particles (62%) and after refinement and postprocessing resulted in a map at 3.61 Å. Three rounds of per-particle CTF refinement in Relion3[8] improved resolution to 3.37 Å.

To check for conformational heterogeneity, we performed 3D classifications without imposed C3 symmetry at different stages of image processing, and we did not find other conformations present. We also did 3D classifications of individual protomers after symmetry expansion and signal subtraction to check for conformational heterogeneity within the trimer. All particles were clustered in one class indicating the presence of only one protein conformation within the trimer. Bayesian polishing in RELION3[35] did not lead to further improvement in maps resolution. The resolution was estimated using the 0.143 cut-off criterion[36] with gold-standard Fourier shell correlation (FSC) between two independently refined half-maps[37]. During post-processing, the approach of high-resolution noise substitution was used to correct for convolution effects of real-space masking on the FSC curve[38]. The directional resolution anisotropy of density map was quantitatively evaluated using the 3DFSC web interface (https://3dfsc.salk.edu)[39].

Cryo-EM model building and validation

Models were built in COOT[40] using the previously determined ASCT2 structure[41] in detergent as reference. The resolution of the maps was of a good quality to unambiguously assign the protein sequence and model most of the residues (47–489). Blurring of the final maps to b-factors -100 Å^2 and -50 Å^2 helped to control loops fitting. We note that the tip of HP2 (G434 - A426) was poorly resolved.

The empirically determined “ligand up” orientation was different from the docking pose of *Lc*-BPE in the ASCT2 model (“ligand down”; Fig. S8A). The homology model of ASCT2 guided alternative orientations of S354, D464, and C467 sidechains allowing fitting “ligand down” conformation of the *Lc*-BPE in the observed cryo-EM density. Indeed, the refined cryo-EM structure for the “ligand down” ligand binding conformation obtained an improved enrichment score (AUC of 84.6 and logAUC 44.9, compared to AUC 69.5 and logAUC 32.4), supporting the remodeling (Fig. S8D).

Real-space refinements were performed in Phenix[42] with NCS restrains option. The quality of the fit was validated by a Fourier shell cross correlation (FSCsum) between the refined model and the final map. To monitor the effects of potential overfitting, random shifts (up to 0.5 Å) were introduced into the coordinates of the final model, followed by refinement against the first unfiltered half map. The FSC between this shaken-refined model and the first half map used during validation refinement is termed FSCwork, and the FSC against the second half map, which was not used at any point during refinement, is termed FSCfree. A marginal gap between the curves describing FSCwork and FSCfree indicates no overfitting of the model. The SBGrid software package tool was used[43]. Images were prepared with PyMOL[6], ChimeraX[43], and Chimera[17].

Synthesis

Chemicals were purchased from VWR or Sigma-Aldrich. Except for one isomer, all other compounds were synthesized following the same general procedure as shown in the reaction scheme.

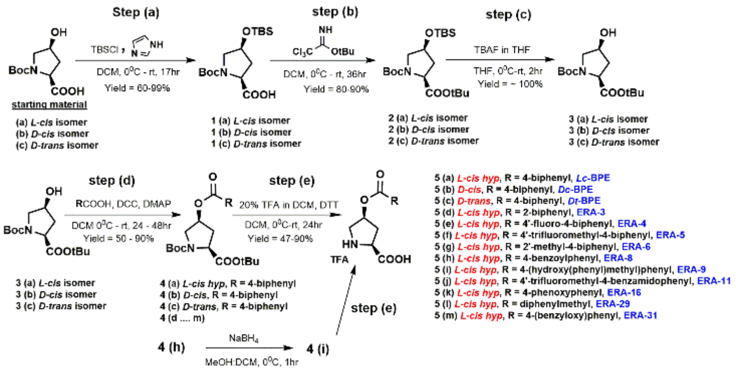

Scheme 1: **General synthesis of D- and L- hydroxyproline ester derivatives**.
(a) TBSCl, imidazole, DCM, 17h, yield 99%. (b) tert-Butyl 2,2,2-trichloroacetimidate, DCM, 36 h, yield 80-90%. (c) TBAF, THF, 2h, yield ~ 100% (d) Aryl carboxylic acid,

DCC, DMAP, DCM, 24 - 48h, total yield 50 - 90%. (e) DTT, 20% TFA in DCM, yield 60 - 90%.

Scheme 2: synthesis of A, L– trans hydroxyproline and B, L– serine ester

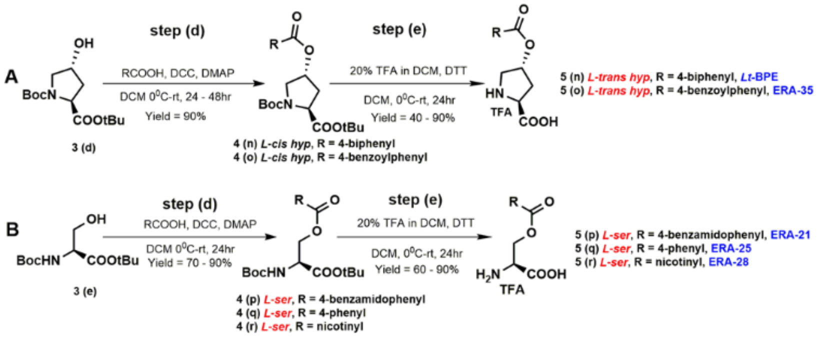

derivatives.

(d) Aryl carboxylic acid, DCC, DMAP, DCM, 24 - 48h, yield 90%. (e) DTT, 20% TFA in DCM, yield 60%.

General synthesis procedures: Scheme 1 and Scheme 2

Step (a) Scheme 1: (tert-butoxycarbonyl)-4-hydroxypyrrolidine-2-carboxylic acid **(a)**, **(b)** or **(c),** (500 mg, 2.16 mmol) and imidazole (744 mg, 10.80 mmol) were weighed into an oven-dried round bottomed flask (RBF) and dissolved in dry DMF (6 mL). The reaction mixture was cooled to 0 ^{0}C and TBSCl in dry DMF (652 mg, 4.32 mmol) was added dropwise under N2 gas. The reaction mixture was then left to warm up to room temperature and stirred for 24 hours. After removal of excess DMF using N2 gas at 50 ^{0}C, the residue was suspended in ethyl acetate and washed twice with water, thrice with chilled 1 M HCl and once with brine. The organic layer was dried over anhydrous Na2SO4 and filtered. The filtrate was concentrated under reduced pressure to a colorless oil. The oil was dissolved in methanol (3 mL) and THF (4 mL) and the solution cooled to 0 ^{0}C. LiOH·H2O (227 mg, 5.40 mmol) in water (3 mL) was added dropwise and the mixture was allowed to warm to room temperature and stirred for 2 hours. The pH of the solution was adjusted to 2-3 using chilled 1 M HCl and the product was collected as a pure, white precipitate after suction filtration.

Step (b) Scheme 1: (*tert*-butoxycarbonyl)-4-((*tert*-butyldimethylsilyl)oxy)pyrrolidine-2-carboxylic acid **1 (a)**, **1 (b)** or **1 (c),** (661 mg, 1.91 mmol) was dissolved in dry DCM and cooled to 0 ^{0}C. 1.03 mL of *tert*-Butyl 2,2,2-trichloroacetimidate (1,252 mg, 5.73 mmol) was added dropwise under N2 and the mixture was stirred for 36 hours at room temperature. Excess solvent was removed *in vacuo* and the residue purified using flash silica gel chromatography (0 – 15% ethyl acetate in hexanes) to obtain a pure colorless oil.

Step (c) Scheme 1: di-*tert*-butyl 4-((*tert*-butyldimethylsilyl)oxy)pyrrolidine-1,2-dicarboxylate **2 (a)**, **2 (b)** or **2 (c),** (635 mg, 1.58 mmol) was dissolved in dry THF and cooled to 0 ^{0}C. 1M TBAF in THF (2.05 mL, 2.05 mmol) was added dropwise under N2 atmosphere and reaction left to stir for 2 hours. Contents were diluted 25% ethyl acetate

in hexanes and washed saturated NH4Cl (1x), chilled 0.5M HCl (1x), NaHCO3 (1x) and 1:1 mixture of H2O and brine (1x). The organic layer was dried over Na2SO4 and filtered. The filtrate was concentrated *in vacuo* and the colorless oil formed was used in the next step without further purification. For **3 (i)** L – *cis* isomer, the product was purified using flash silica gel chromatography (25 – 60% ethyl acetate in hexanes) to obtain a colorless oil which was precipitated from DCM with hexanes to obtain a pure white solid.

Step (d) Scheme 1 & 2: di-*tert*-butyl 4-hydroxypyrrolidine-1,2-dicarboxylate **3 (a)**, **3 (b) 3 (c), 3 (d)**, or tert-butyl (tert-butoxycarbonyl)-L-serinate **3 (e),** (0.18 mmol, 1.0 equiv), DMAP (4 mg, 0.04 mmol), [1,1'-biphenyl]-4-carboxylic acid (144 mg, 0.72 mmol) were dissolved in dry DCM and cooled to 0 ^{0}C. DCC in dry DCM (75, 0.36 mmol) was added dropwise under N2 gas and the reaction mixture was allowed to warm up to room temperature and was stirred for 48 hours. The contents were filtered off and the filtrate concentrated *in vacuo*. The residue was suspended in 50% ethyl acetate in hexanes and washed with NaHCO3 (3x), chilled 0.5M HCl (3x), brine (3x) and H2O (1x). The organic phase was collected and dried over Na2SO4 and filtered. The filtrate was concentrated *in vacuo* and the product purified using flash silica gel chromatography (10 – 50% ethyl acetate in hexanes) to obtain a pure white solid.

Step (e) Scheme 1 & 2: 4 (a), **4 (b) 4 (c), 4 (d)** or **4 (e),** (0.16 mmol, 1.0 equiv) and DTT (49 mg, 0.32 mmol) were weighed into a 10 mL long-necked RBF and dissolved in dry DCM. Trifluoroacetic acid (TFA) (0.39 mL, 5.12 mmol) was added dropwise under N2 gas and the reaction mixture was stirred at room temperature for 36 hours. TFA was completely removed under reduced pressure and the product was collected as a white solid after trituration in methanol and diethyl ether. The product was confirmed by TLC (20 - 40% methanol in DCM), rf ~ 0.2) and was used in all experiments without further purification.

NMR data for major intermediates

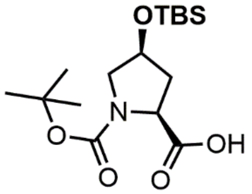

(2S,4S)-1-(tert-butoxycarbonyl)-4-((tert-butyldimethylsilyl)oxy)pyrrolidine-2-carboxylic acid **1 (a).** Synthesized according to general procedure for step (a) in scheme 1. Yield 70%, white solid. ^{1}H NMR (Chloroform-*d*, 400 MHz) δ 11.06 (1H, s), 4.52 – 4.10 (2H, m), 3.58 (1H, d, *J*=14.5 Hz), 3.47 – 3.10 (1H, m), 2.20 (2H, dd, *J*=61.1, 26.1 Hz), 1.42 (9H, s), 0.84 (9H, s), 0.05 (6H, s). ^{13}C NMR (Chloroform-*d*, 101 MHz) δ 176.91, 154.12, 80.77, 70.21, 58.06, 54.48, 39.26, 28.41, 25.72, 18.02, -4.87.

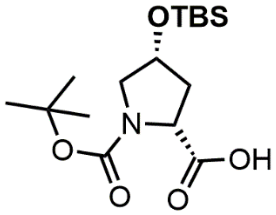

(2R,4R)-1-(tert-butoxycarbonyl)-4-((tert-butyldimethylsilyl)oxy)pyrrolidine-2-carboxylic acid **(1 (b)**. Prepared following general procedure step (a) in scheme 1. Yield 60%, white solid. ^{1}H NMR (Chloroform-*d*, 400 MHz) δ 10.24 (1H, s), 4.39 (2H, d, *J*=22.7 Hz), 3.68 – 3.18 (2H, m), 2.21 (2H, s), 1.48 (9H, s), 0.89 (9H, s), 0.11 (6H, s). Full ^{13}C NMR could not be obtained due to poor resolution of carbonyl carbons for major experiments done such as routine ^{13}C NMR and HMBC.

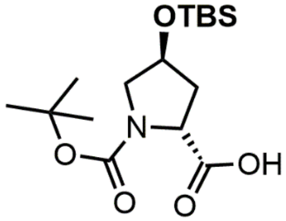

(2R,4S)-1-(tert-butoxycarbonyl)-4-((tert-butyldimethylsilyl)oxy)pyrrolidine-2-carboxylic acid **1 (c).** Compound was synthesized following the general procedure step (a) in scheme 1. Yield 99%, white solid**.** ^{1}H NMR (Chloroform-*d*, 400 MHz) δ 9.71 (1H, s), 4.56 – 4.24 (2H, m), 3.71 – 3.27 (2H, m), 2.41 – 2.15 (1H, m), 2.09 (1H, tt, *J*=8.5, 4.5 Hz), 1.44 (9H, d, *J*=20.8 Hz), 0.87 (9H, s), 0.06 (6H, d, *J*=2.2 Hz). ^{13}C NMR (Chloroform-*d*, 101 MHz) δ 175.07, 157.13, 81.78, 70.13, 58.66, 55.11, 37.79, 28.50, 25.83, 18.07, -4.70.

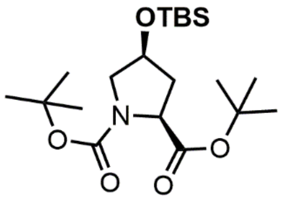
(2S,4S)-di-tert-butyl 4-((tert-butyldimethylsilyl)oxy)pyrrolidine-1,2-dicarboxylate **2 (a)**. Synthesized used general procedure for step (b) in scheme 1. Yield 80%, clear colorless oil. ^{1}H NMR (Chloroform-*d*, 400 MHz) δ 4.27 (1H, h, *J*=5.7 Hz), 4.11 (1H, ddd, *J*=28.2, 8.8, 5.6 Hz), 3.62 (1H, ddd, *J*=38.5, 10.9, 6.1 Hz), 3.18 (1H, ddd, *J*=19.5, 10.9, 5.0 Hz), 2.31 (1H, dddd, *J*=18.9, 12.9, 8.8, 5.9 Hz), 1.91 (1H, dt, *J*=13.0, 5.5 Hz), 1.46 – 1.35 (18H, m), 0.82 (9H, d, *J*=4.4 Hz), 0.00 (6H, s). ^{13}C NMR (Chloroform-*d*, 101 MHz) δ 171.31, 153.90, 80.84, 79.77, 69.77, 58.34, 53.98, 39.45, 28.41, 28.11, 25.84, 18.17.

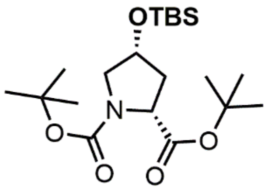
(2R,4R)-di-tert-butyl4-((tert-butyldimethylsilyl)oxy)pyrrolidine-1,2-dicarboxylate **2 (b)**. Compound was synthesized as 2 (b) above following step (b) in scheme 1 general procedure. Yield 86%, clear colorless oil. ^{1}H NMR (Chloroform-*d*, 400 MHz) δ 4.29 (1H, h, *J*=5.6 Hz), 4.13 (1H, ddd, *J*=28.5, 8.8, 5.6 Hz), 3.64 (1H, ddd, *J*=39.5, 10.9, 6.1 Hz), 3.21 (1H, ddd, *J*=19.4, 10.9, 5.1 Hz), 2.44 – 2.23 (1H, m), 1.93 (1H, dt, *J*=13.1, 5.5 Hz), 1.51 – 1.37 (18H, m), 0.85 (9H, d, *J*=4.3 Hz), 0.03 (6H, s). ^{13}C NMR (Chloroform-*d*, 101 MHz) δ 171.38, 153.96, 80.92, 79.85, 69.81, 58.40, 54.01, 39.50, 28.46, 28.16, 25.89, 18.23

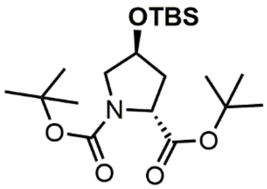
(2R,4S)-di-tert-butyl 4-((tert-butyldimethylsilyl)oxy)pyrrolidine-1,2-dicarboxylate **2 (c)**. Compound was synthesized following step (b) in scheme 1 general procedure. Yield 90%, clear colorless oil. ^{1}H NMR (Chloroform-*d*, 400 MHz) δ 4.40 (1H, p, *J*=5.0 Hz), 4.23 (1H, ddd, *J*=31.4, 8.3, 6.0 Hz), 3.57 (1H, ddd, *J*=16.5, 10.9, 5.2 Hz), 3.42 – 3.17 (1H, m), 2.24 – 2.06 (1H, m), 1.99 (1H, dt, *J*=12.8, 5.4 Hz), 1.53 – 1.38 (18H, m), 0.86 (9H, s), 0.05 (6H, d, *J*=1.7 Hz). ^{13}C NMR (Chloroform-*d*, 101 MHz) δ 172.26, 154.06, 80.94, 79.83, 69.57, 58.75, 54.27, 39.79, 28.34, 28.01, 25.72, 18.02.

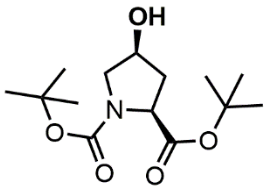 *(2S,4S)-di-tert-butyl 4-hydroxypyrrolidine-1,2-dicarboxylate* **3 (a)**. Synthesized according to step (c) in scheme 1 general procedure. Yield 99%, white solid formed by trituration of colorless oil residue in DCM and hexanes. ^{1}H NMR (Chloroform-*d*, 400 MHz) δ 4.27 (1H, dt, *J*=9.5, 4.5 Hz), 4.15 (1H, ddd, *J*=21.1, 9.8, 1.8 Hz), 3.78 – 3.41 (3H, m), 2.26 (1H, dddd, *J*=20.4, 14.3, 9.8, 4.8 Hz), 2.10 – 1.94 (1H, m), 1.44 (9H, d, *J*=2.5 Hz), 1.41 (9H, d, *J*=4.8 Hz). ^{13}C NMR (Chloroform-*d*, 101 MHz) δ 174.21, 153.94, 82.30, 80.29, 70.30, 58.83, 55.65, 38.77, 28.41, 27.97.

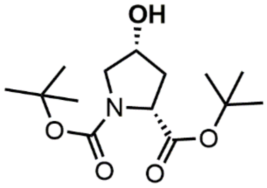 *(2R,4R)-di-tert-butyl 4-hydroxypyrrolidine-1,2-dicarboxylate* **3 (b)**. Synthesized as compound 3 (a) above. Yield 99%, white solid. ^{1}H NMR (Chloroform-*d*, 400 MHz) δ 4.29 (1H, t, *J*=4.4 Hz), 4.25 – 4.02 (1H, m), 3.78 – 3.34 (3H, m), 2.27 (1H, dddd, *J*=20.2, 14.4, 10.5, 4.7 Hz), 2.08 – 1.92 (1H, m), 1.47 (9H, d, *J*=2.2 Hz), 1.43 (9H, d, *J*=4.2 Hz). ^{13}C NMR (Chloroform-*d*, 101 MHz) δ 173.26, 152.87, 81.31, 79.26, 69.33, 57.79, 54.69, 37.71, 27.35, 26.92.

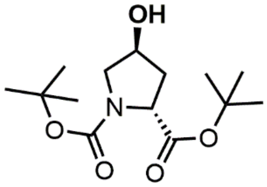

(2R,4S)-di-tert-butyl 4-hydroxypyrrolidine-1,2-dicarboxylate **3 (c)**. Compound synthesized according to general procedure for step (c) in scheme 1. Yield, quantitative (100%). Colorless oil which turned solid on freezing. ^{1}H NMR (Chloroform-*d*, 400 MHz) δ 4.28 (1H, p, *J*=3.8 Hz), 4.13 (1H, q, *J*=7.6, 7.1 Hz), 4.02 (1H, s), 3.49 – 3.21 (2H, m), 2.26 – 2.00 (1H, m), 1.86 (1H, ddd, *J*=12.8, 7.2, 5.0 Hz), 1.39 – 1.22 (18H, m). ^{13}C NMR (Chloroform-*d*, 101 MHz) δ 172.19, 154.20, 80.96, 80.02, 68.67, 58.52, 54.38, 38.96, 28.20, 27.85.

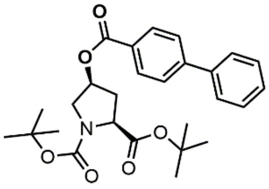

(2S,4S)-di-tert-butyl 4-(([1,1'-biphenyl]-4-carbonyl)oxy)pyrrolidine-1,2-dicarboxylate **4 (a)**. Synthesized according to Steglich esterification general procedure step (d) in scheme 1. Yield 90%, white solid. ^{1}H NMR (Chloroform-*d*, 400 MHz) δ 8.07 (2H, dd,

J=8.3, 5.1 Hz), 7.61 (4H, td, *J*=8.3, 7.4, 2.0 Hz), 7.44 (2H, t, *J*=7.5 Hz), 7.38 (1H, t), 5.52 (1H, ddt, *J*=7.6, 5.7, 2.3 Hz), 4.38 (1H, ddd, *J*=47.5, 9.6, 2.1 Hz), 3.93 – 3.62 (2H, m), 2.56 (1H, dddd, *J*=20.4, 14.8, 9.7, 5.4 Hz), 2.46 – 2.31 (1H, m), 1.47 (9H, d, *J*=8.0 Hz), 1.41 (9H, d, *J*=3.8 Hz). ^{13}C NMR (Chloroform-*d*, 101 MHz) δ 170.76, 165.92, 153.90, 145.91, 139.99, 130.44, 128.98, 128.52, 128.23, 127.32, 126.98, 81.20, 80.09, 72.48, 58.52, 52.36, 36.75, 28.40, 28.08.

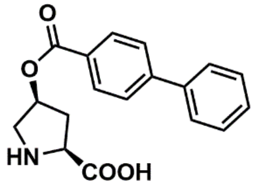

(2S,4S)-4-(([1,1'-biphenyl]-4-carbonyl)oxy)pyrrolidine-2-carboxylic acid **5 (a)**,.***Lc*-BPE**. Prepared according to general TFA deprotection procedure step (e) in scheme 1. **4 (a)** (140 mg, 0.30 mmol), DTT (94 mg, 0.60 mmol) and TFA (0.73 mL, 9.60 mmol). Product details; 60 mg, 47% as an off-white solid

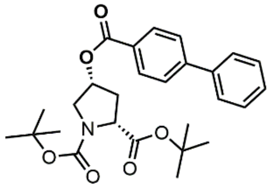

(2R,4R)-di-tert-butyl 4-(([1,1'-biphenyl]-4-carbonyl)oxy)pyrrolidine-1,2-dicarboxylate **4 (b)**. Compound was synthesized following step (d) general procedure in scheme 1. Yield 91%, white solid. ^{1}H NMR (Chloroform-*d*, 400 MHz) δ 8.08 (2H, dd, *J*=8.2, 4.8 Hz), 7.62 (4H, td, *J*=7.9, 1.3 Hz), 7.46 (2H, t, *J*=7.5 Hz), 7.43 – 7.36 (1H, m), 5.53 (1H, ddt, *J*=7.6, 2.1 Hz), 4.39 (1H, ddd, *J*=47.8, 9.7, 2.1 Hz), 3.94 – 3.61 (2H, m), 2.57 (1H, dddd, *J*=21.1, 14.8, 9.7, 5.4 Hz), 2.48 – 2.25 (1H, m), 1.48 (9H, d, *J*=8.4 Hz), 1.41 (9H, d, *J*=4.1 Hz). ^{13}C NMR (Chloroform-*d*, 101 MHz) δ 170.86, 166.05, 153.99, 146.02, 140.12, 130.53, 129.07, 128.59, 128.31, 127.42, 127.09, 81.32, 80.22, 72.55, 58.61, 52.42, 36.85, 28.49, 28.17.

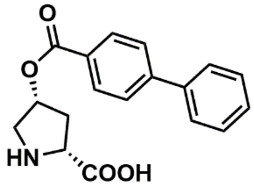

(2R,4R)-4-(([1,1'-biphenyl]-4-carbonyl)oxy)pyrrolidine-2-carboxylic acid **5 (b)**,.***Dc*-BPE**. Prepared according to general TFA deprotection procedure step (e) in scheme 1. **4 (b)** (71 mg, 0.15 mmol), DTT (47 mg, 0.30 mmol) and TFA (0.50 mL, 4.80 mmol). Product details; 40 mg, 62% as an off-white solid

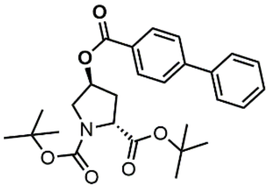

(2R,4S)-di-tert-butyl 4-(([1,1'-biphenyl]-4-carbonyl)oxy)pyrrolidine-1,2-dicarboxylate **4 (a)**. Synthesized using general procedure (step d). Yield 95%, white solid. ^{1}H NMR (Chloroform-*d*, 400 MHz) δ 8.16 – 7.96 (2H, m), 7.73 – 7.57 (4H, m), 7.47 (2H, t, *J*=7.5 Hz), 7.40 (1H, d, *J*=7.3 Hz), 5.52 (1H, tt, *J*=5.0, 2.7 Hz), 4.38 (1H, dt, *J*=32.0, 7.7 Hz), 3.95 – 3.54 (2H, m), 2.68 – 2.42 (1H, m), 2.41 – 2.19 (1H, m), 1.48 (18H, d, *J*=8.1 Hz). ^{13}C NMR (Chloroform-*d*, 101 MHz) δ 171.75, 165.95, 154.04, 146.13, 140.00, 130.32, 129.07, 128.58, 128.36, 127.40, 127.22 (d, *J*=2.4 Hz), 81.59 (d, *J*=3.6 Hz), 80.49, 72.68, 58.73 (d, *J*=3.4 Hz), 52.18, 36.97, 28.46, 28.15.

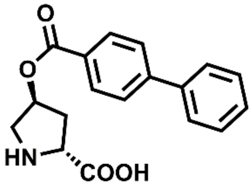
(2R,4S)-4-(([1,1'-biphenyl]-4-carbonyl)oxy)pyrrolidine-2-carboxylic acid **5 (c)**,.***Dt*-BPE**. Prepared according to general TFA deprotection procedure step (e) in scheme 1. **4 (c)** (74 mg, 0.16 mmol), DTT (49 mg, 0.32 mmol) and TFA (0.50 mL, 5.06 mmol). Product details; 63 mg, 94% as a white powder.

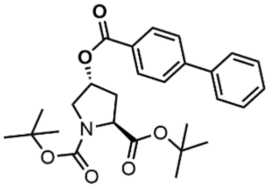

(2S,4R)-di-tert-butyl 4-(([1,1'-biphenyl]-4-carbonyl)oxy)pyrrolidine-1,2-dicarboxylate **4 (n)**. Synthesized according to scheme 2 step (d) general procedure in scheme 2 (B). Yield 90%. Yellow solid. ^{1}H NMR (Chloroform-*d*, 600 MHz) δ 8.10 – 8.04 (2H, m), 7.65 (2H, dd, *J*=8.2, 3.4 Hz), 7.63 – 7.58 (2H, m), 7.48 – 7.43 (2H, m), 7.42 – 7.36 (1H, m), 5.52 (1H, ddt, *J*=7.7, 2.4 Hz), 4.38 (1H, dt, *J*=49.0, 7.7 Hz), 3.91 – 3.64 (2H, m), 2.62 – 2.45 (1H, m), 2.31 (1H, dddd, *J*=15.3, 10.2, 7.4, 5.5 Hz), 1.48 (18H, d, *J*=12.3 Hz). 13C NMR (Chloroform-d, 151 MHz) δ 171.72, 165.90, 154.02, 146.09, 139.94 (d, J=5.5 Hz), 130.28, 129.03, 128.55, 128.32, 127.36, 127.16, 81.54, 80.47, 72.65, 58.68, 52.15, 36.93, 28.43, 28.12.

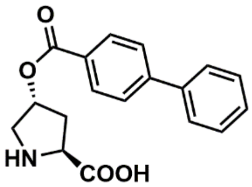
(2S,4R)-4-(([1,1'-biphenyl]-4-carbonyl)oxy)pyrrolidine-2-carboxylic acid **5 (n)**,.***Lt*-BPE**. Prepared according to general TFA deprotection procedure step (e) in scheme 1. **4 (n)** (90 mg, 0.19 mmol), DTT (60 mg, 0.39 mmol) and TFA (0.47 mL, 6.14 mmol). Product details; 74 mg, 90 % as a white solid.

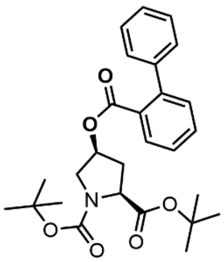

di-tert-butyl (2S,4S)-4-(([1,1'-biphenyl]-2-carbonyl)oxy)pyrrolidine-1,2-dicarboxylate **4 (d)**. Synthesized according to Mitsunobu esterification protocol. **3 (d)** (100 mg, 0.35 mmol), biphenyl-2-carboxylic acid (138 mg, 0.70 mmol) and diphenyl(4-pyridyl)phosphine (183 mg, 0.70 mmol) were dissolved in dry THF (1.0 mL) and cooled to 0^0C. TEA (0.1 mL, 0.70 mmol) and DIAD (0.15 mL, 0.70 mmol) added under N2 atmosphere. The reaction was left to warm up to room temperature and stirred for 24 hours monitored by TLC. Reaction mixture was transferred into separating funnel and diluted with EtOAc and washed with 0.5M HCl (10 mL x3), NaHCO3 (10 mL x3) and brine (5 mL x3). The organic layer was dried over Na2SO4 and concentrated in vacuo. The residue was purified using silica gel flash chromatography using 0 - 25% EtOAc in hexanes to obtain a pure colorless oil (37 mg, 23 %) and impure fraction 120 mg (not used). The pure fraction was used in the next step.
^{1}H NMR (Chloroform-*d*, 400 MHz) δ 7.89 – 7.72 (1H, m), 7.38 (7H, dq, *J*=13.0, 7.8 Hz), 7.29 (2H, dp, *J*=5.1, 1.7 Hz), 5.15 (1H, tt, *J*=5.8, 2.7 Hz), 4.23 (1H, ddd, *J*=37.1, 9.4, 2.8 Hz), 3.64 (1H, ddd, *J*=17.6, 12.4, 5.7 Hz), 3.24 (1H, ddd, *J*=53.7, 12.5, 2.6 Hz), 2.35 (1H, dddd, *J*=38.3, 14.7, 9.4, 5.7 Hz), 1.97 (1H, ddd, *J*=16.6, 5.5, 2.8 Hz), 1.44 (9H, d, *J*=13.1 Hz), 1.30 (9H, d, *J*=6.7 Hz)
^{13}C NMR (Chloroform-*d*, 101 MHz) δ 170.83, 168.10, 153.86, 142.93, 141.35, 131.57, 130.68, 129.98, 128.67, 128.52, 128.24, 127.36, 81.26, 80.07, 72.48, 58.38, 51.81, 35.76, 28.49, 27.93

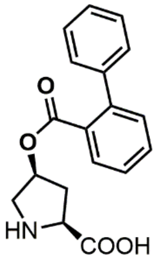

(2S,4S)-4-(([1,1'-biphenyl]-2-carbonyl)oxy)pyrrolidine-2-carboxylic acid **5 (d)**,.**ERA-3**. Prepared according to general TFA deprotection procedure step (e) in scheme 1. **4 (d)** (37 mg, 0.08 mmol), DTT (25 mg, 0.16 mmol) and TFA (0.2 mL, 0.19 mmol). Product details; 20 mg, 60% as a white precipitate.

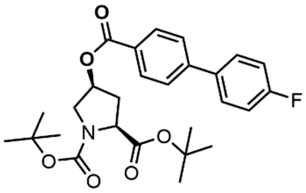
di-tert-butyl (2S,4S)-4-((4'-fluoro-[1,1'-biphenyl]-4-carbonyl)oxy)pyrrolidine-1,2-dicarboxylate **4 (e)**. Synthesized according to the general procedure step (e) in scheme 1. **3 (a)** (40 mg, 0.14 mmol), DMAP (2 mg, 0.01 mmol), 4'-fluoro-[1,1'-biphenyl]-4-carboxylic acid (90 mg, 0.42 mmol) and DCC (43, 0.21 mmol). Product details; 60 mg, 89% as a white solid

^{1}H NMR (Chloroform-*d*, 400 MHz) δ 8.06 (2H, dd, *J*=8.3, 4.6 Hz), 7.59 – 7.52 (4H, m), 7.14 (2H, t, *J*=8.6 Hz), 5.52 (1H, dp, *J*=5.6, 2.0 Hz), 4.38 (1H, ddd, *J*=47.8, 9.7, 2.1 Hz), 3.92 – 3.57 (2H, m), 2.57 (1H, dddd, *J*=21.0, 14.9, 9.7, 5.4 Hz), 2.46 – 2.33 (1H, m), 1.47 (9H, d, *J*=8.3 Hz), 1.40 (9H, d, *J*=10.1 Hz)

^{13}C NMR (Chloroform-*d*, 101 MHz) δ 170.83, 165.93, 153.98, 144.94, 136.19, 130.57, 129.05, 126.89, 81.28, 80.22, 72.59, 58.48, 52.63, 36.81, 28.54, 28.14

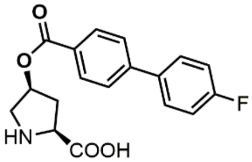
(2S,4S)-4-((4'-fluoro-[1,1'-biphenyl]-4-carbonyl)oxy)pyrrolidine-2-carboxylic acid **5 (e)**,.**ERA-4**. Prepared according to general TFA deprotection procedure step (e) in scheme 2. **4 (e)** (52 mg, 0.11 mmol), DTT (33 mg, 0.21 mmol) and TFA (1.0 mL, excess). Product details; 35 mg, 74% as an off-white powder.

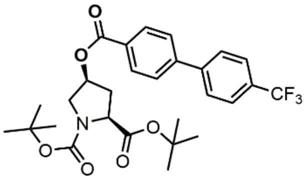

di-tert-butyl (2S,4S)-4-((4'-(trifluoromethyl)-[1,1'-biphenyl]-4-carbonyl)oxy)pyrrolidine-1,2-dicarboxylate **4 (f).** Synthesized according to the general protocol step (d) in scheme 1. **3 (a)** (35 mg, 0.12 mmol), DMAP (3 mg, 0.02 mmol), 4'-(trifluoromethyl)-[1,1'-biphenyl]-4-carboxylic acid (130 mg, 0.49 mmol) and DCC (50, 0.24 mmol). Product details; 58 mg, 88% as a white solid

^{1}H NMR (Chloroform-*d*, 400 MHz) δ 8.10 (2H, dd, *J*=8.3, 4.8 Hz), 7.71 (4H, m), 7.65 – 7.63 (2H, m), 5.62 – 5.43 (1H, m), 4.39 (1H, ddd, *J*=47.4, 9.7, 2.0 Hz), 3.92 – 3.62 (2H, m), 2.71 – 2.48 (1H, m), 2.47 – 2.30 (1H, m), 1.48 (9H, s), 1.41 (9H, d, *J*=2.9 Hz)

^{13}C NMR (Chloroform-*d*, 101 MHz) δ 170.84, 165.77, 154.37, 153.98, 144.42, 143.61, 142.20, 136.83, 127.75, 127.64 – 127.48 (m), 127.28, 80.25, 72.73, 58.56, 57.66, 32.42, 30.93, 28.46, 28.15

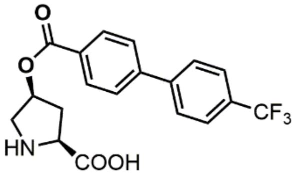

(2S,4S)-4-((4'-(trifluoromethyl)-[1,1'-biphenyl]-4-carbonyl)oxy)pyrrolidine-2-carboxylic acid **5 (f)**,.**ERA-5**. Prepared according to general TFA deprotection procedure step (e) in scheme 1. **4 (f)** (58 mg, 0.11 mmol), DTT (33 mg, 0.22 mmol) and TFA (1.0 mL, excess). Product details; 40 mg, 75% as an off-white powder.

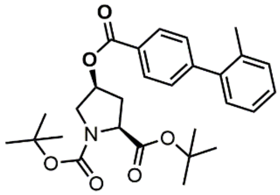

di-tert-butyl (2S,4S)-4-((2'-methyl-[1,1'-biphenyl]-4-carbonyl)oxy)pyrrolidine-1,2-dicarboxylate **4 (g)**. Synthesized according to the general procedure step (d) in scheme 1. **3 (a).** (48 mg, 0.17 mmol), DMAP (2 mg, 0.02 mmol), 2'-methyl-[1,1'-biphenyl]-4-carboxylic acid (106 mg, 0.50 mmol) and DCC (52, 0.25 mmol). Product details; 80 mg, 99% as a white solid.

^{1}H NMR (Chloroform-*d*, 400 MHz) δ 8.04 (2H, dd, *J*=8.1, 4.8 Hz), 7.35 (2H, dt, *J*=8.3, 2.4 Hz), 7.29 – 7.22 (4H, m), 5.53 (1H, tt, *J*=5.4, 2.2 Hz), 4.39 (1H, ddd, *J*=50.3, 9.7, 2.1 Hz), 3.92 – 3.61 (2H, m), 2.56 (1H, dddd, *J*=21.4, 14.7, 9.6, 5.3 Hz), 2.39 (1H, dd, *J*=15.2, 5.4 Hz), 2.22 (3H, s), 1.47 (9H, d, *J*=9.2 Hz), 1.40 (9H, d, *J*=2.6 Hz)

^{13}C NMR (Chloroform-*d*, 101 MHz) δ 170.80, 166.11, 154.06, 147.19, 140.95, 135.22, 130.61, 130.10, 129.84, 129.55, 129.31, 128.26, 80.23, 72.53, 58.60, 52.40, 36.79, 30.86, 28.50, 28.14, 26.34

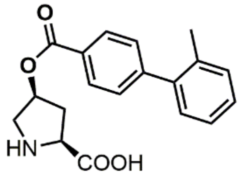

(2S,4S)-4-((2'-methyl-[1,1'-biphenyl]-4-carbonyl)oxy)pyrrolidine-2-carboxylic acid **5 (g)**,.**ERA-6**. Prepared according to general TFA deprotection procedure step (e) in scheme 1. **4 (g)** (80 mg, 0.17 mmol), DTT (51 mg, 0.33 mmol) and TFA (1.0 mL, excess). Product details; 45 mg, 62% as an off-white powder.

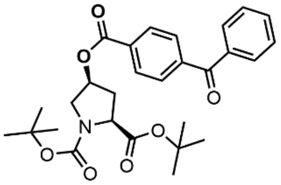

di-tert-butyl (2S,4S)-4-((4-benzoylbenzoyl)oxy)pyrrolidine-1,2-dicarboxylate **4 (h)**. Synthesized according to the general procedure step (d) in scheme 1. **3 (a).** (80 mg, 0.28 mmol), DMAP (6.8 mg, 0.06 mmol), 4-benzoylbenzoic acid (189 mg, 0.84 mmol) and DCC (63, 0.31 mmol). Product details; 120 mg, 87% as a white solid.

^{1}H NMR (Chloroform-*d*, 400 MHz) δ 8.19 – 8.05 (2H, m), 7.88 – 7.72 (4H, m), 7.62 (1H, ddt, *J*=8.9, 7.0, 1.8 Hz), 7.49 (2H, dd, *J*=8.4, 7.0 Hz), 5.55 (1H, tt, *J*=5.1, 2.1 Hz), 4.39

(1H, ddd, *J*=48.3, 9.7, 2.0 Hz), 3.93 – 3.62 (2H, m), 2.58 (1H, dddd, *J*=20.2, 14.8, 9.7, 5.3 Hz), 2.39 (1H, ddd, *J*=14.5, 3.9, 2.1 Hz), 1.47 (9H, d, *J*=10.1 Hz), 1.40 (9H, d, *J*=2.1 Hz)

^{13}C NMR (Chloroform-*d*, 101 MHz) δ 196.16, 170.81, 165.43, 154.02, 141.67, 137.06, 133.11, 132.92, 130.24, 129.86, 128.60, 81.46, 80.32, 73.61, 58.46, 52.67, 36.26, 28.51, 28.18

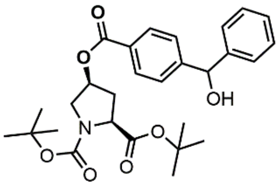

(2S,4S)-4-((4-benzoylbenzoyl)oxy)pyrrolidine-2-carboxylic acid **5 (h)**,.**ERA-8**. Prepared according to general TFA deprotection procedure step (e) in scheme 1. **4 (h)** (116 mg, 0.23 mmol), DTT (72 mg, 0.46 mmol) and TFA (0.60 mL, 7.36 mmol). Product details; 73 mg, 69% as an off-white powder.

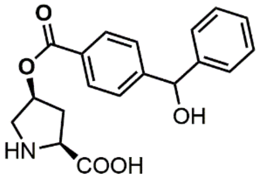

di-tert-butyl (2S,4S)-4-((4-(hydroxy(phenyl)methyl)benzoyl)oxy)pyrrolidine-1,2-dicarboxylate **4 (i)**. Synthesized according to the general procedure step (d) in scheme 1. **4 (h).** (47 mg, 0.09 mmol) was dissolved in DCM (0.5 mL)/methanol 1.0 mL mixture and cooled to 0^{0}C. NaBH4 (7.1 mg, 0.19 mmol) was added in one portion. The reaction let to warm up to room temperature and stirred for 2 hours until complete conversion as monitored by TLC. Reaction was quenched by addition of acetone and transferred into separating funnel and 5.0 mL of water added then product extracted with DCM (10 mL x3). Organic layer was dried over Na2SO4 and filtered off. Filtrate was concentrated in vacuo and residue purified using silica gel flash chromatography to yield 35 mg, 75% as a white solid.

^{1}H NMR (Chloroform-*d*, 400 MHz) δ 7.97 (2H, dd, *J*=8.2, 3.9 Hz), 7.44 (2H, dd, *J*=8.3, 2.2 Hz), 7.33 (4H, d, *J*=3.6 Hz), 7.30 – 7.26 (1H, m), 5.87 (1H, s), 5.48 (1H, dq, *J*=5.7, 2.7 Hz), 4.36 (1H, ddd, *J*=46.5, 9.7, 2.1 Hz), 3.88 – 3.53 (2H, m), 2.55 (1H, dddd, *J*=21.0, 14.8, 9.6, 5.5 Hz), 2.35 (1H, dd, *J*=14.7, 4.2 Hz), 1.46 (9H, d, *J*=8.4 Hz), 1.42 – 1.33 (9H, m)

^{13}C NMR (Chloroform-*d*, 101 MHz) δ 170.81, 165.93, 153.98, 149.09, 143.40, 130.22, 128.90, 128.13, 126.84, 126.38, 81.30, 80.24, 76.06, 36.77, 28.51, 28.15

(2S,4S)-4-((4-(hydroxy(phenyl)methyl)benzoyl)oxy)pyrrolidine-2-carboxylic acid **5 (i)**,.**ERA-9**. Prepared according to general TFA deprotection procedure step (e) in scheme 1. **4 (i)** (35 mg, 0.07 mmol), DTT (22 mg, 0.14 mmol) and TFA (0.2 mL, 2.25 mmol). Product details; 8 mg, 25% as an off-white powder.

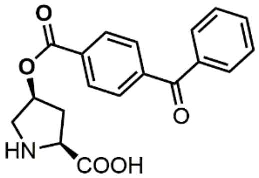

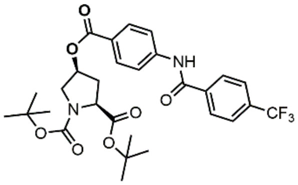

di-tert-butyl (2S,4S)-4-((4-(4-(trifluoromethyl)benzamido)benzoyl)oxy)pyrrolidine-1,2-dicarboxylate **4 (j)**. Synthesized according to the general procedure step (d) in scheme 1. **3 (a).** (54 mg, 0.20 mmol), DMAP (2.5 mg, 0.02 mmol), (4'-Trifluoromethyl)-4-benzamidobenzoic acid (187 mg, 0.60 mmol) and DCC (46, 0.22 mmol). Product details; 48 mg, 41% as colorless oil, white solid on freezing.

^{1}H NMR (Chloroform-*d*, 400 MHz) δ 9.17 – 8.28 (1H, m), 8.00 (4H, q, *J*=8.2, 7.7 Hz), 7.74 (4H, dd, *J*=18.3, 8.1 Hz), 5.46 (1H, s), 4.35 (1H, dd, *J*=29.7, 9.5 Hz), 3.95 – 3.49 (2H, m), 2.71 – 2.43 (1H, m), 2.36 (1H, d, *J*=14.6 Hz), 1.59 – 1.26 (18H, m)

^{13}C NMR (Chloroform-*d*, 101 MHz) δ 170.79, 165.56, 164.95, 154.22, 142.52, 138.02, 131.26, 127.96, 125.78, 119.57, 81.52, 80.43, 73.00, 58.48, 52.65, 36.29, 28.47, 28.14

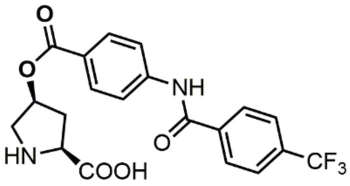

(2S,4S)-4-((4-(4-(trifluoromethyl)benzamido)benzoyl)oxy)pyrrolidine-2-carboxylic acid **5 (j)**,.**ERA-11**. Prepared according to general TFA deprotection procedure step (e) in scheme 1. **4 (j)** (48 mg, 0.08 mmol), DTT (26 mg, 0.17 mmol) and TFA (0.20 mL, 2.66 mmol). Product details; 17 mg, 38% off-white powder.

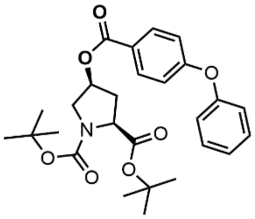

di-tert-butyl (2S,4S)-4-((4-phenoxybenzoyl)oxy)pyrrolidine-1,2-dicarboxylate **4 (k)**. Synthesized according to the general procedure step (d) in scheme 1. **3 (a).** (90 mg, 0.31 mmol), DMAP 19 mg, 0.16 mmol), 4-phenoxybenzoic acid (302 mg, 1.41 mmol) and DCC (97, 0.47 mmol). Product details; 128 mg, 85%, white solid.

^{1}H NMR (Chloroform-*d*, 400 MHz) δ 8.03 – 7.89 (2H, m), 7.47 – 7.31 (2H, m), 7.18 (1H, t, *J*=7.5 Hz), 7.09 – 7.00 (2H, m), 7.00 – 6.83 (2H, m), 5.48 (1H, dtq, *J*=6.2, 4.2, 1.9 Hz), 4.36 (1H, ddd, *J*=47.7, 9.7, 2.1 Hz), 3.91 – 3.59 (2H, m), 2.54 (1H, dddd, *J*=20.8, 14.8, 9.7, 5.4 Hz), 2.35 (1H, ddd, *J*=14.4, 4.1, 2.3 Hz), 1.46 (9H, d, *J*=9.4 Hz), 1.39 (9H, d, *J*=2.3 Hz)

^{13}C NMR (Chloroform-*d*, 101 MHz) δ 170.79, 165.67, 162.11, 155.76, 154.05, 132.15, 130.14, 124.59, 124.26, 120.11, 117.41, 81.35, 80.21, 72.91, 58.46, 52.62, 36.30, 28.50, 28.16

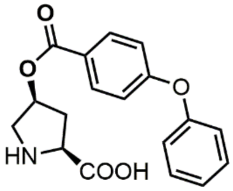

(2S,4S)-4-((4-phenoxybenzoyl)oxy)pyrrolidine-2-carboxylic acid **5 (k)**,.**ERA-16**. Prepared according to general TFA deprotection procedure step (e) in scheme 1. **4 (k)** (57 mg, 0.12 mmol), DTT (36 mg, 0.24 mmol) and TFA (0.30 mL, 3.77 mmol). Product details; 20 mg, 39% as a white solid.

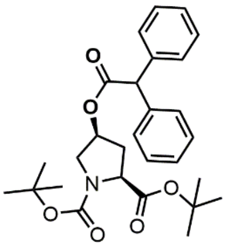

di-tert-butyl (2S,4S)-4-(2,2-diphenylacetoxy)pyrrolidine-1,2-dicarboxylate **4 (l)**.
Synthesized according to the general procedure step (d) in scheme 1. **3 (a).** (100 mg,

0.35 mmol), DMAP 8.5 mg, 0.07 mmol), Diphenylacetic acid (366 mg, 1.72 mmol) and DCC (72, 0.35 mmol). Product details; 154 mg, 92%, clear light green-orange oil .
^{1}H NMR (Chloroform-*d*, 400 MHz) δ 7.39 – 7.19 (10H, m), 5.30 (1H, tdt, *J*=11.2, 8.1, 4.0 Hz), 4.95 (1H, d, *J*=2.1 Hz), 4.29 (1H, ddd, *J*=43.3, 9.5, 2.6 Hz), 3.80 (1H, ddd, *J*=14.9, 12.4, 5.9 Hz), 3.50 (1H, ddd, *J*=36.8, 12.4, 2.6 Hz), 2.48 (1H, dddd, *J*=27.6, 15.0, 9.6, 6.0 Hz), 2.17 (1H, dq, *J*=14.4, 2.6 Hz), 1.46 (9H, d, *J*=6.9 Hz), 1.39 (9H, d, *J*=4.1 Hz)
^{13}C NMR (Chloroform-*d*, 101 MHz) δ 172.13, 170.73, 153.83, 138.40, 138.25, 128.63, 127.41, 81.24, 80.12, 73.01, 58.33, 56.69, 52.24, 35.80, 28.41, 27.97

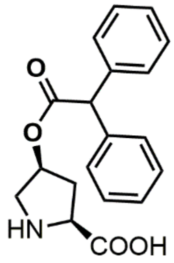

(2S,4S)-4-(2,2-diphenylacetoxy)pyrrolidine-2-carboxylic acid **5 (l)**,.**ERA-29**. Prepared according to general TFA deprotection procedure step (e) in scheme 1. **4 (l)** (154 mg, 0.32 mmol) and TFA (1.76 mL, 23.04 mmol). Product details; 73 mg, 52% as an off-white powder.

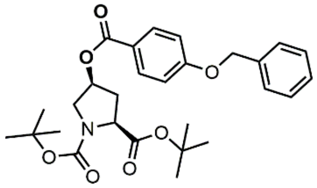

di-tert-butyl (2S,4S)-4-((4-(benzyloxy)benzoyl)oxy)pyrrolidine-1,2-dicarboxylate **4 (m)**. Synthesized according to the general procedure step (d) in scheme 1. **3 (a).** (160 mg, 0.56 mmol), DMAP 34 mg, 0.28 mmol), 4-(benzyloxy)benzoic acid (381 mg, 1.67 mmol) and DCC (126, 0.61 mmol) and TEA (0.16 mL, 2 eq). Procedure modified to enhance solubility of carboxylic acid. Product details; 277 mg, 100%, white solid.
^{1}H NMR (Chloroform-*d*, 400 MHz) δ 7.99 – 7.87 (2H, m), 7.40 – 7.29 (5H, m), 6.93 (2H, dd, *J*=8.9, 2.1 Hz), 5.44 (1H, tt, *J*=5.1, 2.0 Hz), 5.06 (2H, d, *J*=1.7 Hz), 4.34 (1H, ddd, *J*=45.8, 9.7, 2.1 Hz), 3.87 – 3.56 (2H, m), 2.50 (1H, dddd, *J*=20.9, 14.8, 9.7, 5.4 Hz), 2.32 (1H, dq, *J*=14.3, 2.2 Hz), 1.45 (9H, d, *J*=7.6 Hz), 1.37 (9H, s)
^{13}C NMR (Chloroform-*d*, 101 MHz) δ 170.65, 165.65, 162.68, 153.88, 136.17, 131.91, 128.61, 128.15, 127.45, 122.30, 114.38, 81.14, 79.94, 72.58, 70.01, 58.33, 52.45, 36.16, 28.35, 27.99

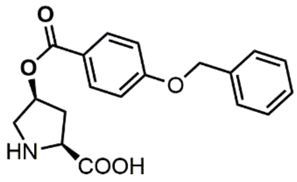

(2S,4S)-4-((4-(benzyloxy)benzoyl)oxy)pyrrolidine-2-carboxylic acid **5 (m)**,.**ERA-31**. Prepared according to general TFA deprotection procedure step (e) in scheme 1. **4 (m)** (250 mg, 0.50 mmol), No DTT used, TFA (2.75 mL, excess). Product details; 127 mg, 56% as a light brown powder.

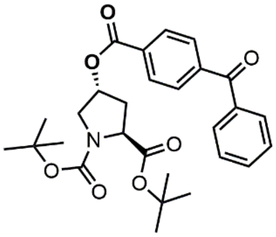

di-tert-butyl (2S,4R)-4-((4-benzoylbenzoyl)oxy)pyrrolidine-1,2-dicarboxylate **4 (o)**. Synthesized according to the general procedure step (d) in scheme 2. (A) **3 (d).** (100 mg, 0.35 mmol), DMAP 8.5 mg, 0.07 mmol), 4-benzoylbenzoic acid (236 mg, 1.04 mmol) and DCC (79, 0.38 mmol). Product details; 190 mg, 100%, colorless oil which turns to white solid on cooling

^{1}H NMR (Chloroform-*d*, 400 MHz) δ 8.07 (2H, dd, *J*=8.4, 2.3 Hz), 7.85 – 7.70 (4H, m), 7.63 – 7.53 (1H, m), 7.45 (2H, dd, *J*=8.4, 7.0 Hz), 5.50 (1H, tt, *J*=5.2, 2.6 Hz), 4.34 (1H, dt, *J*=25.5, 7.7 Hz), 3.92 – 3.58 (2H, m), 2.62 – 2.42 (1H, m), 2.29 (1H, ddd, *J*=13.7, 7.3, 5.2 Hz), 1.44 (18H, d, *J*=11.1 Hz)

^{13}C NMR (Chloroform-*d*, 101 MHz) δ 195.64, 171.32, 165.02, 153.88, 141.51, 136.70, 132.86, 132.58, 129.93, 129.61, 129.43, 128.33, 81.37, 80.18, 73.31, 58.40, 52.03, 36.04, 28.20, 27.84

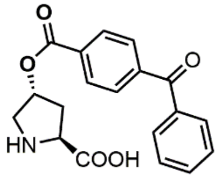

(2S,4R)-4-((4-benzoylbenzoyl)oxy)pyrrolidine-2-carboxylic acid **4 (o)**,.**ERA-35**. Prepared according to general TFA deprotection procedure step (e) in scheme 2, (A). **4 (o)** (100 mg, 0.20 mmol), No DTT used, TFA (2.00 mL, excess). Product details; 82 mg, 90% as a light pink powder.

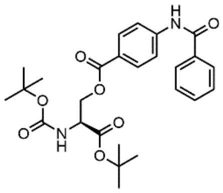

(S)-3-(tert-butoxy)-2-((tert-butoxycarbonyl)amino)-3-oxopropyl 4-benzamidobenzoate **4 (p)**. Synthesized according to the general procedure step (d) in scheme 2.(B) **3 (e).** (250 mg, 0.96 mmol), DMAP (58 mg, 0.48 mmol), 4-benzamidobenzoic acid (923 mg, 3.83 mmol), DCC (217, 1.05 mmol), TEA (0.50 mL, 4 eq) and DMF (0.5 mL). Protocol was modified to improve solubility of carboxylic acid. Product details; 250 mg, 54%, off-white powder.

^{1}H NMR (Chloroform-*d*, 400 MHz) δ 8.11 – 7.94 (3H, m), 7.94 – 7.81 (2H, m), 7.74 (2H, d, *J*=8.4 Hz), 7.58 (1H, t, *J*=7.3 Hz), 7.51 (2H, t, *J*=7.5 Hz), 5.39 (1H, d, *J*=7.6 Hz), 4.72 – 4.43 (3H, m), 1.45 (18H, s)

^{13}C NMR (Chloroform-*d*, 101 MHz) δ 168.84, 165.75, 165.45, 155.22, 142.46, 134.50, 132.29, 131.08, 128.95, 127.08, 125.21, 119.20, 82.88, 80.15, 65.42, 53.59, 28.32, 27.96

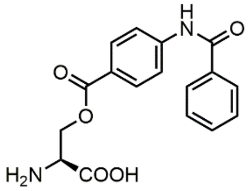

O-(4-benzamidobenzoyl)-L-serine **5 (p)**,.**ERA-21**. Prepared according to general TFA deprotection procedure step (e) in scheme 1. **4 (p)** (140 mg, 0.29 mmol), and TFA (2.0 mL, excess). DDT was not used. Product details; 103 mg, 81%, off-white powder.

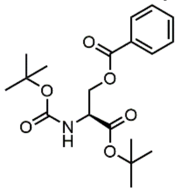

(S)-3-(tert-butoxy)-2-((tert-butoxycarbonyl)amino)-3-oxopropyl benzoate **4 (q)**. Synthesized according to the general procedure step (d) in scheme 2, (B) **3 (e).** (120 mg, 0.46 mmol), DMAP (5.60 mg, 0.05 mmol), benzoic acid (168 mg, 1.38 mmol) and DCC (104 mg, 0.51 mmol). Product details; 149 mg, 88%, white solid.

^{1}H NMR (Chloroform-*d*, 400 MHz) δ 8.04 – 7.95 (2H, m), 7.61 – 7.50 (1H, m), 7.42 (2H, t, *J*=7.7 Hz), 5.39 (1H, d, *J*=7.5 Hz), 4.76 – 4.27 (3H, m), 1.43 (18H, s)

^{13}C NMR (Chloroform-*d*, 101 MHz) δ 168.80, 165.99, 155.17, 133.23, 129.68, 128.42, 82.83, 80.10, 65.42, 53.53, 28.28, 27.92

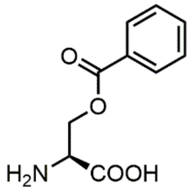

O-benzoyl-L-serine **5 (q)**,.**ERA-25**. Prepared according to general TFA deprotection procedure step (e) in scheme 2, (B). **4 (q)** (50 mg, 0.14 mmol), DDT (42.2 mg, 0.28 mmol) and TFA (0.40 mL, 4.38 mmol). Product details; 24 mg, 53%, collected as a white powder.

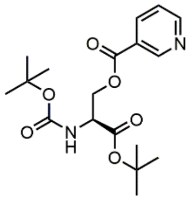

(S)-3-(tert-butoxy)-2-((tert-butoxycarbonyl)amino)-3-oxopropyl nicotinate **4 (r)**. Synthesized using acyl chloride according to the following procedure **3 (e).** (150 mg, 0.58 mmol), DMAP (11 mg, 0.09 mmol) and TEA (1.0 mL, 7.17 mmol) were dissolved in DCM (7.0 mL and solution cooled to 0^0C. Nicotinoyl chloride (123 mg, 0.87) in DCM was added slowly and reaction stirred for 24 hours. Reaction was quenched with 1 mL water and stirred for 30 minutes. Excess solvent was removed in vacuo and residue taken up in 15 mL of 50% EtOAc in hexanes and transferred into separating funnel. Organic layer was washed with chilled 1M HCl (3 x 10 mL), NaHCO3 (3 x 10 mL) and water (1 x 10 mL). The organic layer was dried over Na2SO4, filtered off and filtrated concentrated in vacuo and the residue purified using flash silica gel column chromatography 15% - 40% EtOAc in hexanes to yield pure white powder, 191 mg, 91%.
^{1}H NMR (Chloroform-*d*, 400 MHz) δ 9.17 (1H, s), 8.96 – 8.60 (1H, m), 8.24 (1H, d, *J*=7.9 Hz), 7.37 (1H, dd, *J*=7.9, 4.8 Hz), 5.42 (1H, d, *J*=7.5 Hz), 4.61 (3H, d, *J*=3.7 Hz), 1.42 (18H, d, *J*=5.9 Hz)
^{13}C NMR (Chloroform-*d*, 101 MHz) δ 168.53, 164.65, 155.12, 153.55, 150.83, 137.21, 123.41, 83.06, 80.22, 65.68, 53.46, 28.27, 27.92

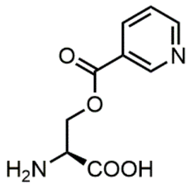

O-nicotinoyl-L-serine **5 (r)**,.**ERA-28**. Prepared according to general TFA deprotection procedure step (e) in scheme 2, (B). **4 (r)** (75 mg, 0.21 mmol), DDT (63 mg, 0.41 mmol)

and TFA (0.80 mL, 6.56 mmol). Product details; 27 mg, 41%, collected as a white powder.

References

1 Albers, T., Marsiglia, W., Thomas, T., Gameiro, A. & Grewer, C. Defining substrate and blocker activity of alanine-serine-cysteine transporter 2 (ASCT2) Ligands with Novel Serine Analogs. *Molecular Pharmacology* **81**, 356-365, doi:10.1124/mol.111.075648 (2012).

2 Grewer, C. & Grabsch, E. New inhibitors for the neutral amino acid transporter ASCT2 reveal its Na+-dependent anion leak. *The Journal of physiology* **557**, 747-759, doi:10.1113/jphysiol.2004.062521 (2004).

3 Davis, I. W. *et al.* MolProbity: all-atom contacts and structure validation for proteins and nucleic acids. *Nucleic Acids Res* **35**, W375-383, doi:10.1093/nar/gkm216 (2007).

4 Canul-Tec, J. C. *et al.* Structure and allosteric inhibition of excitatory amino acid transporter 1. *Nature* **544**, 446-451, doi:10.1038/nature22064 (2017).

5 Garibsingh, R. A. *et al.* Homology Modeling Informs Ligand Discovery for the Glutamine Transporter ASCT2. *Front Chem* **6**, 1-12, doi:10.3389/fchem.2018.00279 (2018).

6 Schrodinger, LLC. *The PyMOL Molecular Graphics System, Version 2.0 Schrödinger, LLC* (2020).

7 Krivov, G. G., Shapovalov, M. V. & Dunbrack, R. L., Jr. Improved prediction of protein side-chain conformations with SCWRL4. *Proteins* **77**, 778-795, doi:10.1002/prot.22488 (2009).

8 Colas, C. *et al.* Ligand Discovery for the Alanine-Serine-Cysteine Transporter (ASCT2, SLC1A5) from Homology Modeling and Virtual Screening. *PLoS Comput Biol* **11**, 1-22, doi:10.1371/journal.pcbi.1004477 (2015).

9 Esslinger, C. S., Cybulski, K. A. & Rhoderick, J. F. Ngamma-aryl glutamine analogues as probes of the ASCT2 neutral amino acid transporter binding site. *Bioorg Med Chem* **13**, 1111-1118, doi:10.1016/j.bmc.2004.11.028 (2005).

10 Schulte, M. L., Khodadadi, A. B., Cuthbertson, M. L., Smith, J. A. & Manning, H. C. 2-Amino-4-bis(aryloxybenzyl)aminobutanoic acids: A novel scaffold for inhibition of ASCT2-mediated glutamine transport. *Bioorg Med Chem Lett* **26**, 1044-1047, doi:10.1016/j.bmcl.2015.12.031 (2016).

11 Singh, K. *et al.* Structure activity relationships of benzylproline-derived inhibitors of the glutamine transporter ASCT2. *Bioorg Med Chem Lett* **27**, 398-402, doi:10.1016/j.bmcl.2016.12.063 (2017).

12 Schulte, M. L., Dawson, E. S., Saleh, S. A., Cuthbertson, M. L. & Manning, H. C. 2-Substituted Nγ-glutamylanilides as novel probes of ASCT2 with improved potency. *Bioorg Med Chem Lett* **25**, 113-116, doi:10.1016/j.bmcl.2014.10.098 (2015).

13 Gaulton, A. *et al.* ChEMBL: a large-scale bioactivity database for drug discovery. *Nucleic acids research* **40**, D1100-1107, doi:10.1093/nar/gkr777 (2012).
14 Mysinger, M. M., Carchia, M., Irwin, J. J. & Shoichet, B. K. Directory of useful decoys, enhanced (DUD-E): better ligands and decoys for better benchmarking. *Journal of medicinal chemistry* **55**, 6582-6594, doi:10.1021/jm300687e (2012).
15 McGann, M. FRED pose prediction and virtual screening accuracy. *J Chem Inf Model* **51**, 578-596, doi:10.1021/ci100436p (2011).
16 Schrodinger, L. Schrodinger Release 2018-4: Glide. In, New York, NY. (2018).
17 Pettersen, E. F. *et al.* UCSF Chimera--a visualization system for exploratory research and analysis. *J Comput Chem* **25**, 1605-1612, doi:10.1002/jcc.20084 (2004).
18 Jo, S., Kim, T., Iyer, V. G. & Im, W. CHARMM-GUI: a web-based graphical user interface for CHARMM. *J Comput Chem* **29**, 1859-1865, doi:10.1002/jcc.20945 (2008).
19 Huang, J. *et al.* CHARMM36m: an improved force field for folded and intrinsically disordered proteins. *Nat Methods* **14**, 71-73, doi:10.1038/nmeth.4067 (2017).
20 Bonomi, M., Pellarin, R. & Vendruscolo, M. Simultaneous Determination of Protein Structure and Dynamics Using Cryo-Electron Microscopy. *Biophys J* **114**, 1604-1613, doi:10.1016/j.bpj.2018.02.028 (2018).
21 Vahidi, S. *et al.* Reversible inhibition of the ClpP protease via an N-terminal conformational switch. *Proc Natl Acad Sci U S A* **115**, E6447-E6456, doi:10.1073/pnas.1805125115 (2018).
22 Abraham, M. J. M., T.; Schulz, R.; Páll, S.; Smith, J. C.; Hess, B.; Lindahl, E. GROMACS: High performance molecular simulations through multi-level parallelism from laptops to supercomputers. *SoftwareX* **1-2**, 19-25 (2015).
23 Bonomi, M. & Camilloni, C. Integrative structural and dynamical biology with PLUMED-ISDB. *Bioinformatics* **33**, 3999-4000, doi:10.1093/bioinformatics/btx529 (2017).
24 Bonomi, M., et al.,. Promoting transparency and reproducibility in enhanced molecular simulations. *Nat Methods* **16**, 670-673, doi:10.1038/s41592-019-0506-8 (2019).
25 Daura, X. *et al.* Peptide Folding: When Simulation Meets Experiment. *Angewandte Chemie International Edition* **38**, 236-240, doi:10.1002/(sici)1521-3773(19990115)38:1/2<236::Aid-anie236>3.0.Co;2-m (1999).
26 Pingitore, P. *et al.* Large scale production of the active human ASCT2 (SLC1A5) transporter in Pichia pastoris--functional and kinetic asymmetry revealed in proteoliposomes. *Biochim Biophys Acta* **1828**, 2238-2246, doi:10.1016/j.bbamem.2013.05.034 (2013).
27 Garaeva, A. A. *et al.* Cryo-EM structure of the human neutral amino acid transporter ASCT2. *Nature structural & molecular biology* **25**, 515-521, doi:10.1038/s41594-018-0076-y (2018).
28 Arkhipova, V., Guskov, A. & Slotboom, D. J. Structural ensemble of a glutamate transporter homologue in lipid nanodisc environment. *Nat Commun* **11**, 1-9, doi:10.1038/s41467-020-14834-8 (2020).
29 Jan Rheinberger, G. O., Guenter P Resch, Cristina Paulino. Optimized data acquisition workflow by sample thickness determination. *bioRxiv* (2020).

30 Biyani, N. *et al.* Focus: The interface between data collection and data processing in cryo-EM. *J Struct Biol* **198**, 124-133, doi:10.1016/j.jsb.2017.03.007 (2017).
31 Zheng, S. Q. *et al.* MotionCor2: anisotropic correction of beam-induced motion for improved cryo-electron microscopy. *Nat Methods* **14**, 331-332, doi:10.1038/nmeth.4193 (2017).
32 Rohou, A. & Grigorieff, N. CTFFIND4: Fast and accurate defocus estimation from electron micrographs. *J Struct Biol* **192**, 216-221, doi:10.1016/j.jsb.2015.08.008 (2015).
33 Punjani, A., Rubinstein, J. L., Fleet, D. J. & Brubaker, M. A. cryoSPARC: algorithms for rapid unsupervised cryo-EM structure determination. *Nat Methods* **14**, 290-296, doi:10.1038/nmeth.4169 (2017).
34 Zivanov, J. *et al.* New tools for automated high-resolution cryo-EM structure determination in RELION-3. *Elife* **7**, 1-22, doi:10.7554/eLife.42166 (2018).
35 Zivanov, J., Nakane, T. & Scheres, S. H. W. A Bayesian approach to beam-induced motion correction in cryo-EM single-particle analysis. *IUCrJ* **6**, 5-17, doi:10.1107/s205225251801463x (2019).
36 Rosenthal, P. B. & Henderson, R. Optimal determination of particle orientation, absolute hand, and contrast loss in single-particle electron cryomicroscopy. *J Mol Biol* **333**, 721-745, doi:10.1016/j.jmb.2003.07.013 (2003).
37 Scheres, S. H. & Chen, S. Prevention of overfitting in cryo-EM structure determination. *Nat Methods* **9**, 853-854, doi:10.1038/nmeth.2115 (2012).
38 Chen, S. *et al.* High-resolution noise substitution to measure overfitting and validate resolution in 3D structure determination by single particle electron cryomicroscopy. *Ultramicroscopy* **135**, 24-35, doi:10.1016/j.ultramic.2013.06.004 (2013).
39 Tan, Y. Z. *et al.* Addressing preferred specimen orientation in single-particle cryo-EM through tilting. *Nat Methods* **14**, 793-796, doi:10.1038/nmeth.4347 (2017).
40 Emsley, P. & Cowtan, K. Coot: model-building tools for molecular graphics. *Acta Crystallogr D Biol Crystallogr* **60**, 2126-2132, doi:10.1107/s0907444904019158 (2004).
41 Yu, X. *et al.* Cryo-EM structures of the human glutamine transporter SLC1A5 (ASCT2) in the outward-facing conformation. *Elife* **8**, 1-17, doi:10.7554/eLife.48120 (2019).
42 Adams, P. D. *et al.* PHENIX: a comprehensive Python-based system for macromolecular structure solution. *Acta Crystallogr D Biol Crystallogr* **66**, 213-221, doi:10.1107/s0907444909052925 (2010).
43 Morin, A. *et al.* Collaboration gets the most out of software. *Elife* **2**, 1-6, doi:10.7554/eLife.01456 (2013).
44 Hille, B. *Ion Channels of Excitable Membranes*. (Sinauer Associates, 2001).
45 Grewer, C., Watzke, N., Wiessner, M. & Rauen, T. Glutamate translocation of the neuronal glutamate transporter EAAC1 occurs within milliseconds. *Proc Natl Acad Sci U S A* **97**, 9706-9711, doi:10.1073/pnas.160170397 (2000).
46 Ndaru, E. *et al.* Novel alanine serine cysteine transporter 2 (ASCT2) inhibitors based on sulfonamide and sulfonic acid ester scaffolds. *J Gen Physiol* **151**, 357-368, doi:10.1085/jgp.201812276 (2019).
